# RNA-Seq Analysis of Human Trigeminal and Dorsal Root Ganglia with a Focus on Chemoreceptors

**DOI:** 10.1371/journal.pone.0128951

**Published:** 2015-06-12

**Authors:** Caroline Flegel, Nicole Schöbel, Janine Altmüller, Christian Becker, Andrea Tannapfel, Hanns Hatt, Günter Gisselmann

**Affiliations:** 1 Department of Cell Physiology, Ruhr-University Bochum, Bochum, Germany; 2 Department of Animal Physiology, Ruhr-University Bochum, Bochum, Germany; 3 Cologne Center for Genomics, University of Köln, Köln, Germany; 4 Institute of Pathology, Ruhr-University Bochum, Bochum, Germany; University of South California, UNITED STATES

## Abstract

The chemosensory capacity of the somatosensory system relies on the appropriate expression of chemoreceptors, which detect chemical stimuli and transduce sensory information into cellular signals. Knowledge of the complete repertoire of the chemoreceptors expressed in human sensory ganglia is lacking. This study employed the next-generation sequencing technique (RNA-Seq) to conduct the first expression analysis of human trigeminal ganglia (TG) and dorsal root ganglia (DRG). We analyzed the data with a focus on G-protein coupled receptors (GPCRs) and ion channels, which are (potentially) involved in chemosensation by somatosensory neurons in the human TG and DRG. For years, transient receptor potential (TRP) channels have been considered the main group of receptors for chemosensation in the trigeminal system. Interestingly, we could show that sensory ganglia also express a panel of different olfactory receptors (ORs) with putative chemosensory function. To characterize OR expression in more detail, we performed microarray, semi-quantitative RT-PCR experiments, and immunohistochemical staining. Additionally, we analyzed the expression data to identify further known or putative classes of chemoreceptors in the human TG and DRG. Our results give an overview of the major classes of chemoreceptors expressed in the human TG and DRG and provide the basis for a broader understanding of the reception of chemical cues.

## Introduction

Detection and processing of environmental chemosensory information is important for living. In addition to gustation and olfaction, the trigeminal system represents a third chemical sense and is provided by the *Nervus trigeminus*. The nasal cavity is innervated by the ophthalmic and maxillary branches of the trigeminal nerve. The trigeminal ganglia (TG) are located at the base of the scull and represent the cranial analogue of the spinal nerve-associated ganglia, the dorsal root ganglia (DRG). The cell bodies of the pseudounipolar TG and DRG neurons can terminate as free nerve endings in the facial skin and mucosae or the dermatomes of the trunk and extremities, respectively. The DRG efferents terminate in the dorsal horn of the spinal cord, and the TG efferents terminate in specific brainstem nuclei.

Somatosensory neurons in the TG and DRG act as sensors for various innocuous and noxious physical and chemical stimuli. Somatosensory neurons of the TG and DRG can serve as the detectors for a warning system in mammals. Sensations transmitted by the somatosensory system encompass temperatures ranging from freezing cold to painfully hot, stinging and burning elicited by plant compounds and mechanosensations such as touch, stroking, and itch [1,2]. We previously reported that the perception of astringency is also a trigeminal sensation [3].

On the molecular level, several classes of membrane receptors and ion channels are known to be critical for the chemosensory capacity of somatosensory neurons [2,4–12]. Central players involved in the detection of chemical cues by TG and DRG neurons include transient receptor potential (TRP) channels, two-pore potassium (K_2_P) channels, and acid-sensing ion channels (ACCN). These channels act as sensors of temperature, mechanical and chemical stimuli, and they are critical for nociception [13,11,5,2]. In addition to ion channels, G protein-coupled receptors (GPCRs) are essential for the detection of a large variety of chemicals [14]. These proteins are the largest superfamily of cell surface proteins and have seven transmembrane domains [15]. They can be activated by either exogenous ligands, such as odorants or tastants, or endogenous ligands, such as neurotransmitters or hormones; thus they play major roles in physiological and pathophysiological processes [16,17]. Known sensory ganglia-associated GPCRs are the mas-related G protein-coupled receptors (MRGPRs), which are, for example, involved in non-histaminic itch [18,19].

It is known that nearly all odorants, particularly at high concentrations, activate intranasal trigeminal nerve fibers and trigeminally innervated respiratory epithelia [20,21]. Odorant activation leads to chemically evoked activity patterns in the TG and higher order neurons in the brain [22–24]. Most odorant molecules simultaneously stimulate both, the olfactory and the trigeminal system within the nasal cavity. Anosmic patients can detect and discriminate highly concentrated volatile odorants, supporting the idea that the trigeminal system essentially contributes to odor detection and discrimination [20,25,1,26]. Despite several studies which focused on odorant detection by the trigeminal system, the principles of molecular odor reception by TG neurons are only rudimentarily explored. Several studies showed that a variety of different odorants at high concentrations activate rodent TRP channels [27–30]. For example, mammalian TRPV1 is weakly activated by vanillin, helional, heliotropyl acetone, citral, geraniol, thymol, and eugenol [27,31–33,30], TRPV3 is activated by monoterpenoids [28], and TRPM8 is activated by linalool, geraniol, hydroxycitronellal and others [27]. Nevertheless, it is likely that additional unidentified trigeminal receptors exist that provide the perception of volatile cues. Our previous sequencing analysis of mouse somatosensory ganglia supports the idea that olfactory receptors (ORs) are expressed in murine sensory ganglia [34]; thus, we focused our chemoreceptor analysis of the TG and DRG on ORs.

To date, no systematic expression analysis of known or potential chemoreceptors expressed in human sensory ganglia is available, and expression analyses of human TG and DRG tissue samples are rare. The expression profiles of TG and DRG for different ion channels or GPCRs have mostly been validated and investigated in rodents [34,35,18,36]. Therefore, we generated and analyzed RNA-Seq data from several human TG and DRG samples. We established the first systematic gene expression profile of the human TG and DRG with a focus on the major classes of chemoreceptors. We subsequently performed microarray analysis, semi-quantitative RT-PCR (semi qRT-PCR), and immunohistochemical staining of human TG and DRG tissue to validate the expression profile of the chosen ORs.

## Results and Discussion

### General

In our study, RNA-Seq data from human TG and DRG samples were generated using the Illumina sequencing technique. We generated sequencing data for four individual human TG samples (TG 1–4) and a pool of 21 human DRG (Table 1). The data were analyzed with TopHat and Cufflinks software, and reads were mapped to the human reference genome (hg19). These tools are widely accepted and were often used in high resolution transcriptome studies. Cufflinks software normalized reads to account for the length of the gene and the depth of sequencing and ensures comparability of different datasets [37,38]. The quantitative expression values were calculated for each sample based on the number of fragments per kilobase of exon per million fragments mapped (FPKM) [37]. The expression values for all genes in every tissue sample are listed in S1 Table. On a rough scale, >0.1 FPKM corresponds to a very weak expression level, 1 FPKM to a weak expression level, 10 FPKM represents a moderate expression level and 100 FPKM indicates a high expression level. As a basis for comparison, we calculated the FPKM values for typical housekeeping genes. For example, the strongly expressed ß-actin gene yielded an expression value between ~1,000–3,000 FPKM whereas the weakly to moderately expressed TATA box binding protein (TBP) was detected at ~5–9 FPKM (S1 Fig). For an overview of the FPKM values for the expression of all genes, we plotted a histogram for the FPKM value distribution for the TG 3 sample (S2 Fig). Expression analysis revealed ~17,000 genes in the appropriate tissue (FPKM >0.1 out of ~23,000 genes). Gene expression generally reflects the relative abundance of a given gene on protein level, but may not always reflect it. RNA-Seq is an appropriate method to establish an overview for the expression of different receptor and has been used very often [39–43,34,38,44]. For further investigations of receptors at the cellular resolution level, in situ hybridization or immunohistochemistry of whole tissue slices might be an additional approach to localize the expression of transcripts or proteins and would indicate whether there are specialized cell types or areas that highly express gene transcripts. We analyzed the transcriptomes of human TG and DRG to assess the expression of chemoreceptor genes in these ganglia. As RNA from the complete ganglia was used for the RNA-Seq experiments, no distinction could be made between different cell types e.g., the neurons and satellite cells that represent the most abundant cell types in sensory ganglia [45]. Thus, even if the total tissue FPKM levels were low, a single transcript’s level may be high in specific cell types. An example of this scenario was shown for the trace amine-associated receptor TAAR1 in brain [46] and for ORs in the olfactory epithelium [42]. The datasets were compared to existing data from a selected panel of human reference tissues (brain, colon liver, lung, skeletal muscle, and testis) [41] and datasets from murine sensory ganglia [34] and provides a basis for further investigations of molecular reception in human sensory ganglia. We confirmed the expression of chemoreceptors which are known to be expressed in sensory ganglia such as TRP or KCNK channels and our study allowed the comparison of different chemoreceptor classes for the first time in human sensory ganglia.

**Table 1:** Sequencing details of RNA-Seq data-sets.

Sample	Organism	Read length [nt]	Read structure	Total prepared [million]	Reads with at least one reported alignment [%]
TG 1	human	36	paired-end	37.7	left: 83.1right: 80.1
TG 2	human	36	single	51.4	71.9
TG 3	human	101	paired-end	33.7	left: 91.6right: 90.3
TG 4	human	101	paired-end	32.5	left: 89.1 right: 91.3
DRG	human	75	single	20.1	88.4

### Olfactory Receptors (ORs)

With approximately 400 functional genes and 600 non-functional pseudogenes in humans, ORs form the largest superfamily of GPCRs [47,48]. Most OR genes consist of ~330 amino acids encoded by an intron-free reading frame of approximately 1,000 nucleotides [49–51]. Initially, it was postulated that ORs, which detect volatile odorant molecules from the environment, are exclusively expressed in the olfactory epithelium, where they are located in the cilia of olfactory sensory neurons [49]. In 1992, one year after the discovery of ORs, Parmentier et al. found that mammalian OR genes are also expressed in a non-olfactory tissue (testis) by PCR [52]. Since then, a growing number of studies have shown OR expression in several non-olfactory human tissues [41,53,54] and described different physiological functions for these ectopically expressed ORs [55–60].

**Fig 1:**
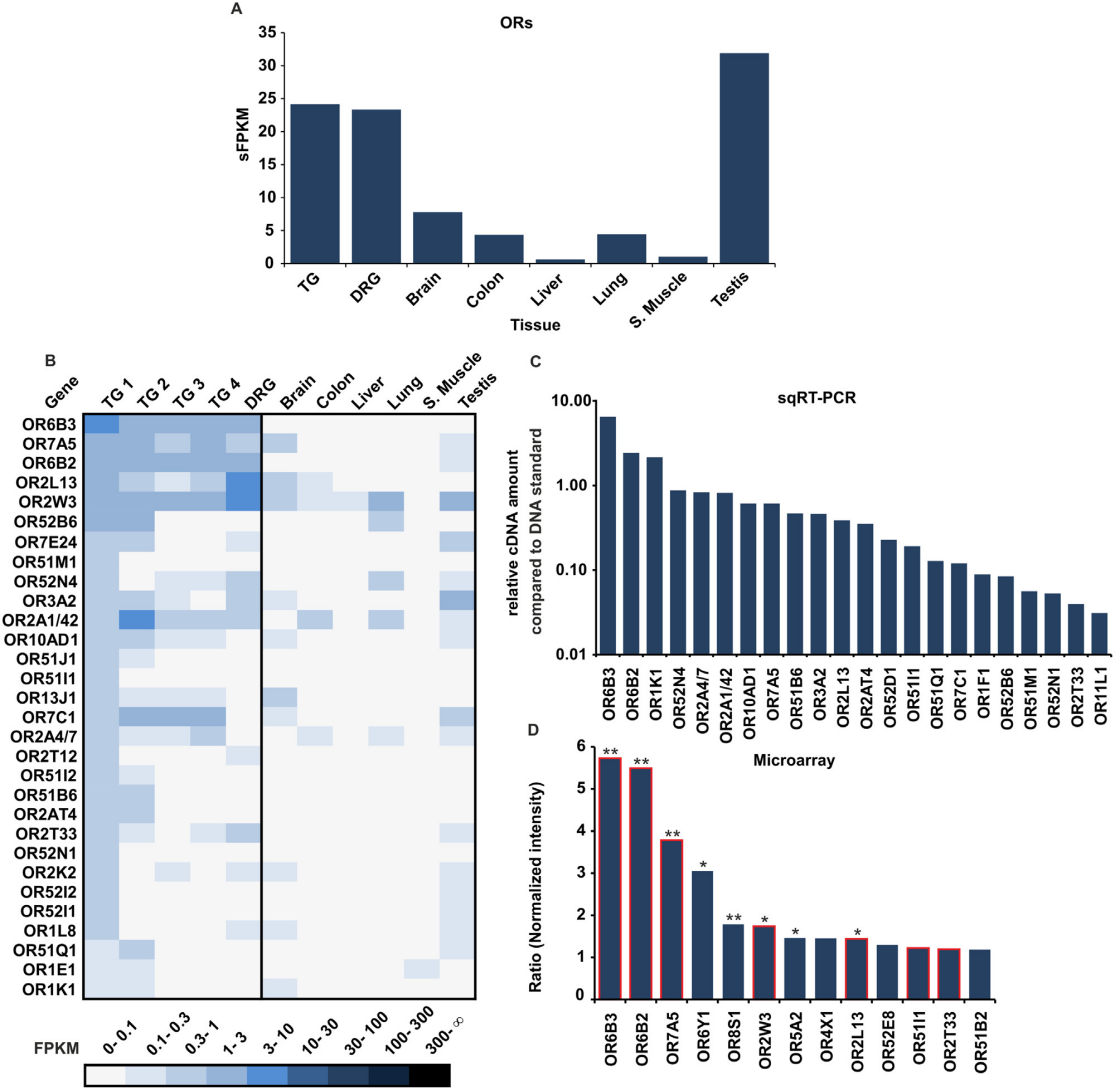
OR expression in the human TG and DRG. **A** The bar diagram shows the summarized expression (sFPKM) of detected OR transcripts. The sFPKM values of OR gene transcripts in the TG and DRG are in the same range as the summarized expression values found in testis tissue. **B** The heatmap shows FPKM values for the 30 most abundant OR transcripts found in the TG 1 sample compared with other TG and DRG samples and reference tissues (brain, colon, liver, lung, s.muscle, and testis). Darker colors indicate higher FPKM values, and white indicates the absence of detectable transcripts. The ORs were sorted according to the FPKM values found in the TG 1 sample. As described in [41], the FPKM values of OR2A4 and OR2A7 as well as that of OR2A1 and OR2A42, which share high sequence similarity (99–100%), were analyzed together. **C** Validation of RNA-Seq results using semi qRT-PCR. The diagram shows the summarized semi qRT-PCR compared with 3 ng of genomic DNA. We investigated 22 different ORs that showed either high, moderate, or low expression in TG 1. **D** Microarray analysis of the ORs in TG 1. Intensity values for the TG 1 sample were normalized to the reference tissues (liver, heart, s. muscle, skin). Given are all ORs with ratios >1.5. ORs were also detected by RNA-Seq as indicated by red frames. t-test: *p<0.05; **p<0.00013 (after Bonferroni correction).

We analyzed the expression of ORs in human TG and DRG samples in a comparative approach using RNA-Seq, microarray analysis, and semi qRT-PCR with a focus on TG 1 sample (Fig 1A). The summarized FPKM values (sFPKM) of the expressed OR genes in the TG (mean sFPKM value of samples TG 1–4) and DRG show that the overall OR expression levels in the TG and DRG are considerably higher (~ 24 sFPKM for TG and ~23 sFPKM for DRG) than in other tissues and comparable to OR expression level in testis (~32 sFPKM) (Fig 1A). Fig 1B shows the OR expression pattern detected by RNA-Seq sorted by the OR expression of the TG 1 sample. Out of the 58 detected ORs in TG 1 (FPKM >0.1), the 30 most highly expressed ORs in comparison to further sensory ganglia and different reference tissues is shown. Based on FPKM values, the RNA for most ORs is present in relatively low abundance compared with that of the housekeeping gene TBP. Out of the 387 annotated OR genes, we detected on average 48 different OR transcripts in a single TG sample and 26 in the DRG sample (FPKM >0.1, S3 Fig). A total of 10 OR transcripts showed FPKM values higher than 1 (OR6B3, OR7A5, OR6B2, OR2L13, OR2W3, OR52B6, OR2A1/42, OR7C1, OR4F21, and OR52H1), and 3 of these transcripts showed values higher than 3 (OR6B3, OR2L13, OR2W3) in one or more tissue samples. The five highest FPKM levels were found for OR2W3 in DRG 1 (6.8 FPKM), OR6B3 in TG 1 (5.2 FPKM), OR2L13 in DRG 1 (3.9 FPKM), OR7A5 in TG 1 (2.6 FPKM), and OR6B2 in TG 1 (2.5 FPKM). The TG and DRG exhibited coherent OR expression patterns and the highest OR transcript levels were consistent for both the TG and DRG. In total, 11 ORs were present in all TG samples, and 39 were detected in at least 2 different TG samples indicating a similar variation and pattern of ORs expressed as already described for human olfactory epithelia [61]. Interestingly, some trigeminally expressed ORs, e.g. OR2W3, were also broadly expressed in several other human tissues [41]. According to our data, some OR transcripts were exclusively present in the TG and DRG (OR6B3). In addition to sensory ganglion tissue, the transcripts of OR6B2 and OR2L13 were weakly detected in testis, colon, or brain. The idea of TG- and DRG-selective receptors is supported by recent RNA-Seq- and PCR-based analyses of the human olfactory epithelium [62,61]. These studies also confirmed that the abovementioned putative TG- and DRG-selective ORs are not or are only weakly expressed in human olfactory epithelium.

We selected 22 ORs that were detectable in the TG 1 RNA-Seq data and confirmed all of the ORs using semi qRT-PCR. The expression levels of ORs determined by PCR correlated with those obtained by RNA-Seq (Fig 1C).

For a long time, microarray was the most commonly used method for transcriptome analysis. Despite the low levels of OR mRNA, microarrays were sufficiently sensitive for detecting ORs in our samples (Fig 1D). Using microarray, we identified 13 ORs with higher expression levels in TG 1 than that in reference tissues (normalized intensities >1.5). The microarray datasets validated the expression of the 5 most highly expressed ORs detected by RNA-Seq (Fig 1D). Furthermore, the microarray data suggested that, in addition to the abovementioned ORs, OR6Y1, OR8S1, OR5A2, OR4X1, OR52E8, and OR51B2 were expressed at higher levels in TG 1 than in reference tissues (Fig 1D). These receptors were not detected by NGS. However, only OR8S1 could be validated by RT-PCR (data not shown), indicating the relatively low reliability of the microarray assay for detecting rare transcripts.

**Fig 2:**
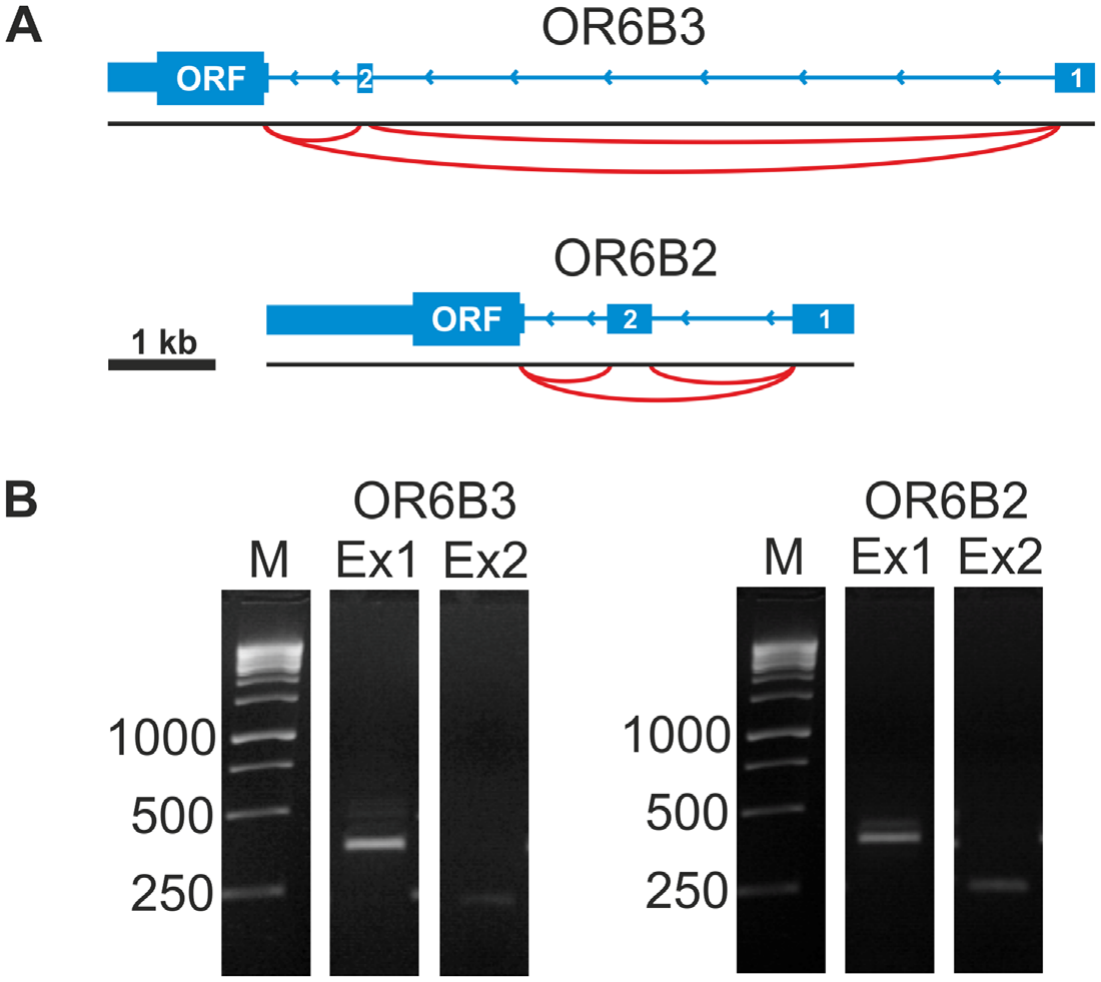
Analysis of highly expressed OR genes in the human TG and DRG. **A** Schematic representation of the newly identified 5’UTRs of OR6B3 and OR6B2. The gene is indicated by blue bars (exon) and thin lines (intron). The coding exon is indicated by ORF (open reading frame) and splice junctions as red arcs. We detected two unannotated 5’UTRs for OR6B3 and OR6B2. **B** 5’UTR-validation of OR-transcripts using RT-PCR with intron-spanning primers in the DRG sample. The newly identified 5’UTRs of the OR transcripts were confirmed by RT-PCR with a forward primer located in the identified exon and a reverse primer located in the ORF of the respective OR. OR6B3: Ex1 (forward primer in exon 1 of 5’UTR and reverse in OR6B3 ORF); Ex2 (forward primer in exon 2 of 5’UTR and reverse primer in OR6B3 ORF). OR6B2: Ex1 (forward primer in exon 1 of 5’UTR and reverse primer in OR6B2 ORF); Ex2 (forward primer in exon 2 of 5’UTR and reverse primer in OR6B2 ORF). The amplified PCR products were confirmed by Sanger sequencing.

In addition, we analyzed the mapped reads in the RNA-Seq data for the 5 most highly expressed OR genes using the Integrative Genomic Viewer (IGV) [63]. For the highly expressed OR genes OR6B3 and OR6B2, we observed aligned sequences in known exons, and we additionally identified intron-spanning reads that connect newly identified 5′ untranslated gene regions (5′UTRs) with open reading frame (ORF)-containing exons (Fig 2A). For OR2W3, we also detected previously described chimeric transcripts with the upstream Trim58 gene in the DRG but not in the TG samples [41]. For OR2L13, we detected previously annotated 5’UTRs in the TG and DRG via RNA-Seq. All spliced transcripts could be validated by RT-PCR (Fig 2B and S4 Fig).

For the most highly expressed ORs identified in our study, ligands are only known for OR7A5 (myrac aldehyde and 4-hydroxy-2,5-dimethyl-3(2H)-furanone). This receptor was functionally characterized in human testis and spermatozoa [64,65]. OR7C1 showed reliable expression in all TG samples investigated (FPKM values up to 1.7) but not in the DRG sample. This receptor can be activated by androstadienone [66]. Interestingly, this testosterone metabolite is present in human male secretions such as saliva, sweat, and semen and has been implicated as a putative human pheromone [67,68]. However, androstadienone does not induce a specific response in the human vomeronasal duct in comparison with respiratory epithelium [69].

**Fig 3:**
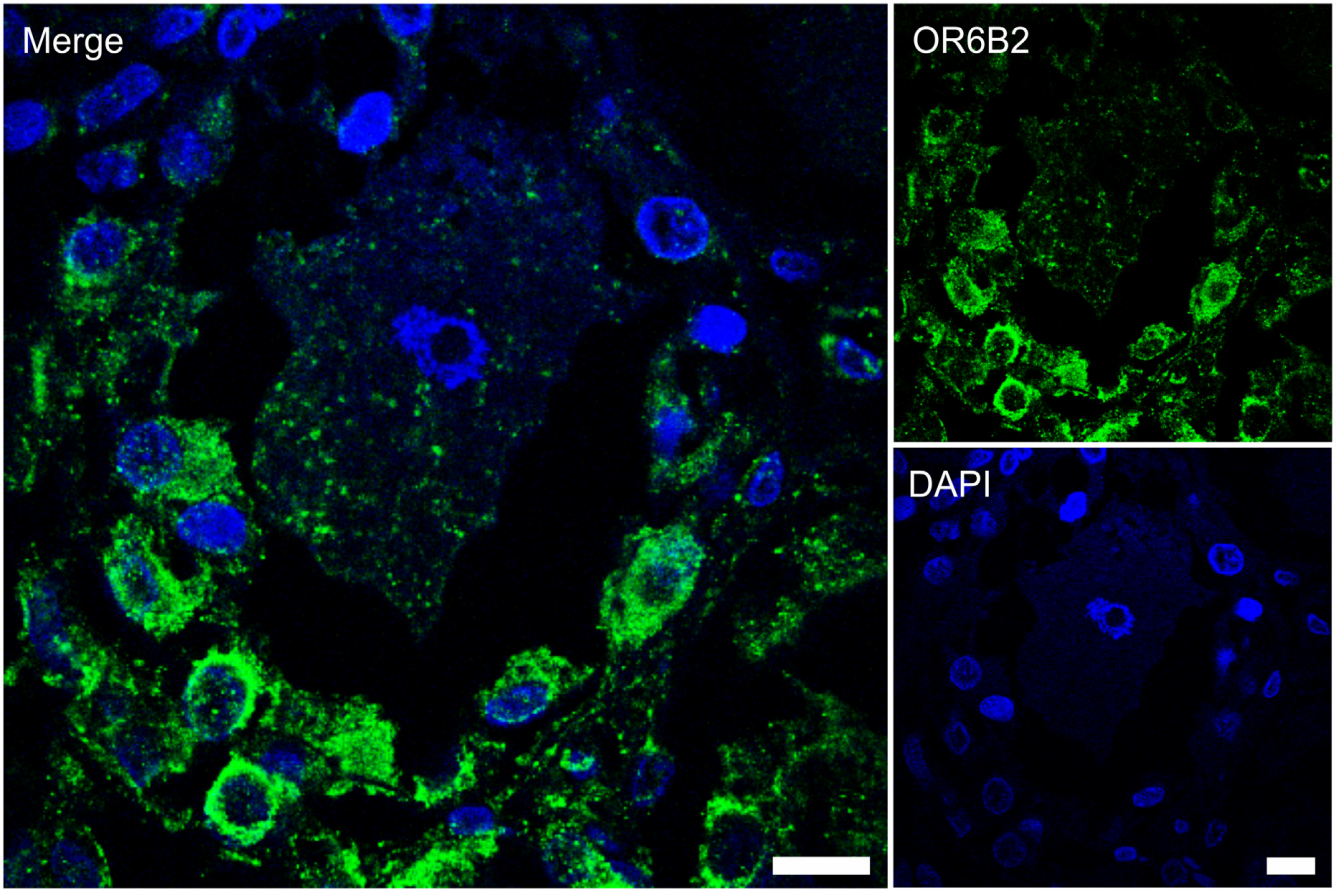
The OR6B2 protein is localized to satellite cells in human DRG. Immunohistochemical staining of human DRG sections with the OR6B2 antibody indicated OR6B2 protein expression in the neuron-surrounding satellite cells. DAPI staining (blue) was used to determine the number and localization of cell nuclei. The secondary antibody alone did not produce any staining (S4 Fig). Scale bars: 10 μm.

To localize cell types in sensory ganglia expressing OR genes, we performed initial immunohistochemical staining (Fig 3). Due to the limited availability of OR antibodies, we used only those targeted against the most highly expressed ORs (OR6B2, OR6B3, OR7A5). However, specificity tests for recombinantly expressed rho-tagged ORs in HANA3A cells showed that only the OR6B2 antibody specifically detects the corresponding OR protein (S5 and S6 Figs), whereas the other OR antibodies were found to be highly nonspecific and thus not used for further experiments. Due to the high sequence homology of OR6B2 and OR6B3 (94%), we cannot exclude that the specific OR6B2 antibody also detects the OR6B3 protein.

Subsequent immunohistochemical staining of human DRG slices indicated that OR6B2-positive cells form a thin envelope around unstained neurons (Fig 3). Stained cells seem to be satellite glial cells that tightly envelop neuronal cell bodies to form discrete anatomical units. As sensory ganglia are not protected from substances circulating in the blood, satellite glial cells may be important in the context of exposure to substances including large molecular weight compounds [70]. For example, it is known that satellite glial cells express ATP receptors. Furthermore, TRPV1 protein expression in satellite glial cells in the rat TG was suggested [71]. Sensory neurons or their surrounding cells monitor blood-borne chemicals, and it was proposed that they might play a chemosensory role on their own [72]. It is tempting to speculate that OR6B2 might be involved in this process but further experiments, such as in situ hybridizations, have to be done to give stronger evidences that ORs are expressed in satellite cells. Due to the limited availability of additional specific OR antibodies and TG slices, the question of which cell type, i.e. neurons or satellite cells within the TG and DRG, express other OR proteins remains to be answered.

This study is the first to show OR transcripts in the human TG and DRG. This subset of specific receptors might contribute to the chemosensory capacity of the TG. In addition, ORs might participate in general peripheral neuronal processes in surrounding satellite cells, which can come in contact with chemical substances via the blood vessels in the DRG and possibly in the TG but further experiments have to be done in the future

### Further chemosensory GPCRs

In addition to ORs, further GPCRs in the TG and DRG bear the potential for detecting chemical cues. Trace amine-associated receptors (TAARs) are non-canonical ORs that are involved in the detection of social cues [73–75]. Recently, it was demonstrated for the first time that human TAAR5 can be activated by amine trimethylamine [76]. TAAR expression is not detectable in the human TG and DRG, and these receptors do not appear to be involved in trigeminal chemosensation (S7 Fig).

The physiological function of human VNO-type chemoreceptors remains elusive. VN1R1 is present in human olfactory epithelium, brain, kidney, liver, and lung [77,78]. Weak expression of VNO-type chemoreceptors can also be detected in sensory ganglia (S8 Fig). Due to their widespread expression throughout the human body, we do not suggest a chemosensory contribution for VNO-type receptors in the human TG and DRG.

Taste receptors are expressed on the tongue where they serve as detectors for different gustatory stimuli. Murine TG and DRG transcriptome analysis exhibited only weak expression of taste receptors in these tissues (FPKM<0.5) [34]. We investigated the expression pattern of all annotated taste receptors in the human TG and DRG via RNA-Seq and detected only a very weak or no expression in human sensory ganglia (S9 Fig). Members of the TAS1R family of taste receptors function as molecular complexes [79]. The heterodimeric TAS1R2/TAS1R3 sweet taste receptor binds sweet stimuli, whereas the TAS1R1/TAS1R3 receptor recognizes amino acids. We detected low transcript levels for the Tas1R3 taste receptor in all sensory ganglia investigated, and for TAS1R1 in 3 of the 4 TG samples and the pool of DRG samples.

Twenty-five different TAS2R genes code for detectors of bitter compounds. Interestingly, it was shown that a variety of bitter taste substances activate rat TG neurons in calcium imaging experiments [80]. In contrast to that, human subjects did not perceive bitterness on the tongue with intact trigeminal innervation after lesion or anesthesia of the taste nerve [3]. However, we found low levels of Tas2R transcripts in sensory ganglia and various other tissues by RNA-Seq. Most detected transcripts of the TAS2R genes lay within the introns of the moderately expressed PRH1-PRR4 gene (S9 Fig). Therefore, the expression of these taste receptor transcripts remains unclear. We suggest that Tas2Rs do not have a specific function in chemosensation in human TG and DRG neurons.

**Fig 4:**
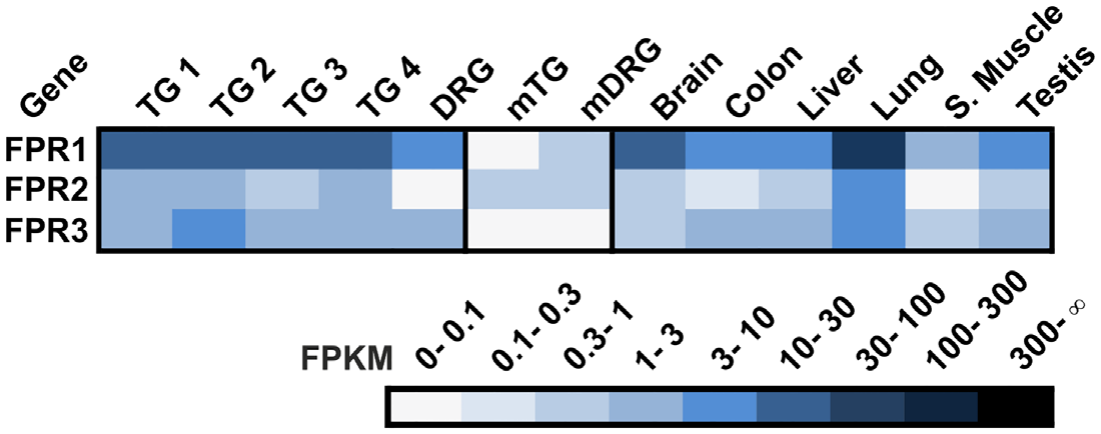
Expression of FPRs in the human TG and DRG. Expression of FPR transcripts could be detected in human (TG and DRG) and murine (mTG and mDRG) sensory ganglia and other reference tissues (brain, colon, liver, lung, s. muscle, and testis).

Formyl Peptide Receptor-like proteins (FPRs) are GPCRs found in all mammals, and they are encoded by three genes in humans [81]. FPRs were attributed an olfactory function associated with the identification of pathogens (bacterial peptides) or the pathogenic state [82]. Fig 4 shows that this group of receptors is highly expressed throughout the human body as well as in murine TG and DRG (mTG and mDRG). In the TG and DRG, the expression of this receptor class has not been described. We could show that FPR1 is highly expressed in the human TG and DRG with FPKM values ≤28. For FPR2 transcripts, we found FPKM values of ≤3 and for FPR3, ≤4 in the sensory ganglia investigated. Only in lung tissue did we detect a higher expression of these three receptors and of FPR1 in the brain. It is tempting to speculate that FPRs may be involved in the trigeminally mediated reflexive stop in inspiration that serves to prevent the inhalation of potentially life-threatening pathogens.

### MRGPR

The family of mas-related G protein-coupled receptors (MRGPRs) counts 8 members with intact coding sequences in humans [35]. In mice, this receptor class is mainly expressed in subpopulations of sensory neurons [35]. Human MRGPR expression has not been investigated in detail, but exclusive MGPRX expression has been shown in human DRG neurons [18]. Although MRGPRX1 and 2 have been detected in human mast cells [83,84], MRGPRs appear to be specifically expressed in sensory neurons. In general, MRGPRs can be activated by peptides [35,18,85] and chemicals [19]. Furthermore, MRGPR-expressing neurons are involved in detecting mechanical or thermal stimuli [86–88].

**Fig 5:**
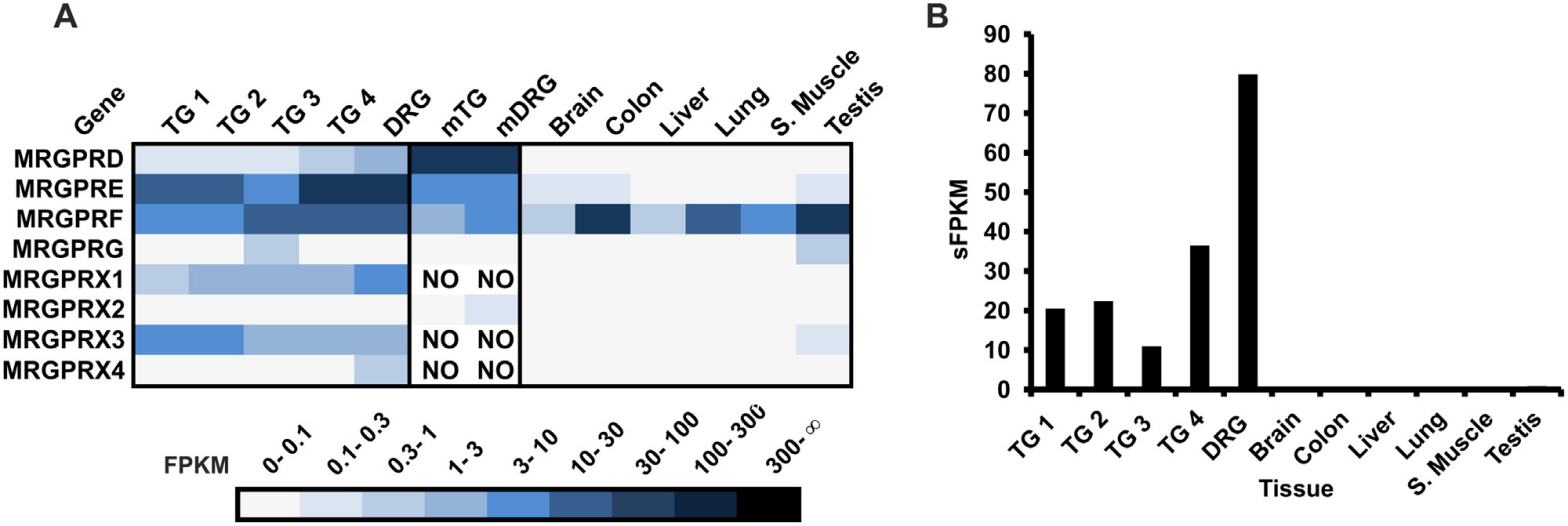
Expression of MRGPRs in the human TG and DRG. **A** MRGPR transcripts were detected in the TG and DRG, whereas they were mostly absent in the reference tissues investigated (brain, colon, liver, lung, s. muscle, and testis). Only MRGPRF transcripts could be detected in all reference tissues. NO = no ortholog **B** Investigation of the sFPKM values of all MRGPRs with the exception of MRGPRF in all tissues. The expression of MRGPRs is restricted to the sensory ganglia investigated.

Our data provide an overview of all MRGPRs expressed in the human TG and DRG in comparison with reference tissues (brain, colon, liver, lung, s.muscle, testis) (Fig 5). RNA-Seq data indicate that the most specific and predominantly expressed MRGPR in human sensory ganglia is MRGPRX1. It is known that this primate-specific MRGPRX1 is enriched in DRG neurons [18] which we can confirm in our datasets of human sensory ganglia (≤9 FPKM in the human TG and DRG) (Fig 5A). MRGPRX1 can be activated by chloroquine and the bovine adrenal medulla peptide BAM8-22, which both induce histamine-independent pruritus [18,19]. Furthermore, MRGPRX1 sensitizes and directly activates TRPV1 via distinct signaling pathways [89]. RNA-Seq data show that MRGPRD is also expressed in the human TG and DRG (≤1.5 FPKM), whereas this receptor is not detectable in human reference tissues (Fig 5A). An involvement of MRGPRD in ß-alanine-mediated pain transmission and its influence on the perception of thermal and mechanical stimuli was suggested [86,87]. Orphan MRGPRE is highly expressed in the human TG and DRG (≤88 FPKM) and nearly absent in reference tissues investigated. We detected high transcript levels for the orphan MRGPRX3 exclusively in the TG and DRG (≤8 FPKM) (Fig 5A). According to our data, MRGPRF is highly expressed in all tissues investigated and therefore the only MRGPR which is also expressed in non-sensory tissues (Fig 5A). In addition, we investigated the distribution of mapped reads in the IGV. We found that human MRGPR transcripts also contain long 3’UTRs as described in mice (S10 Fig).

We show for the first time that in humans most MRGPRs are specifically expressed in the TG and DRG and thus are likely to be involved in somatosensation, e.g. itch and pain (Fig 5B).

### Ion Channels

Several ion channels, including TRP channels, two-pore potassium channels, and calcium-activated chloride channels are directly and indirectly involved in the perception of chemical and physical stimuli by TG and DRG neurons [5,13,90–92]. We examined the expression patterns of different ion channel classes in the human TG and DRG that play a role in chemosensation.

### TRP channels

TRP channels comprise a highly conserved superfamily of cation-permeable ion channels [93]. The TRP superfamily is categorized into 7 subfamilies, TRPC, TRPV, TRPM, TRPML, TRPP, TRPA, and TRPN, of which the latter has not been described in mammals and fish [94]. These channels serve a multitude of physiological functions, including the maintenance of intracellular Ca^2+^ homeostasis and the detection of environmental stimuli. Several TRP channels act as thermosensors, covering a wide range of temperatures, including TRPV1 (> 42°C), TRPV2 (> 52°C), TRPV3 (> 33°C), TRPV4 (25–34°C), TRPA1 (< 17°C), and TRPM8 (< 28°C) [95]. These thermo TRPs include multimodal sensors that also detect either chemical stimuli: TRPV1 (e.g. capsaicin and piperine), TRPV 3 (e.g., thymol and carvacrol), TRPA1 (e.g., cinnamal and allyl-isocyanate), TRPM8 (e.g., menthol and eucalyptol), or mechanical stimulation: TRPV4 [5,96–101,27–30]. The structures, localization, and physiological functions of TRP channels have been intensely studied during the last fifteen years mostly using rodent model organisms. Despite their multiple important somatosensory functions, limited information for TRP channel gene expression in different human tissues, particularly in human somatosensory ganglia is available. TRP channel expression was shown in several human non-chemosensory tissues such as prostate [102], the gastrointestinal system [103–105], eye [106], and brain [107]. The first evidence for functional TRPV1 receptors in human sensory nerves and ganglia was obtained by radioimmunography using [^3^H]-labeled resiniferatoxin, a TRPV1 ligand [108]. More recent studies show immunoreactivity for TRPA1 and TRPV1 in human lingual nerve neuromas [109,110], for TRPV1 in the human tongue [111] and nerve fibers of the nasal mucosa [112], for TRPA1 in the human DRG [113], for TRPV1, TRPV2, and TRPM8 in human skin nerve fibers [114], and for TRPV1, TRPV3, TRPV4, and TRPM8 in the human DRG and skin [115]. In a functional approach, however, human keratinocytes were resistant to vanilloid-mediated stimulation of TRPV1 and showed a much lower TRPV1 expression level than human sensory ganglia [116] suggesting that capsaicin and other TRPV1 agonists act on peripheral nerves but not on keratinocytes in humans. Data on the TRP gene expression or protein immunolocalization in human trigeminal ganglia is nearly absent. In their study, Hou and coworkers showed that VR1 (TRPV1) expression and VR1 immunoreactivity colocalizes with CGRP in human trigeminal tissue [117]. These available fragmentary data indicate not only the presence but also the functional importance of TRP channels in human sensory tissues.

**Fig 6:**
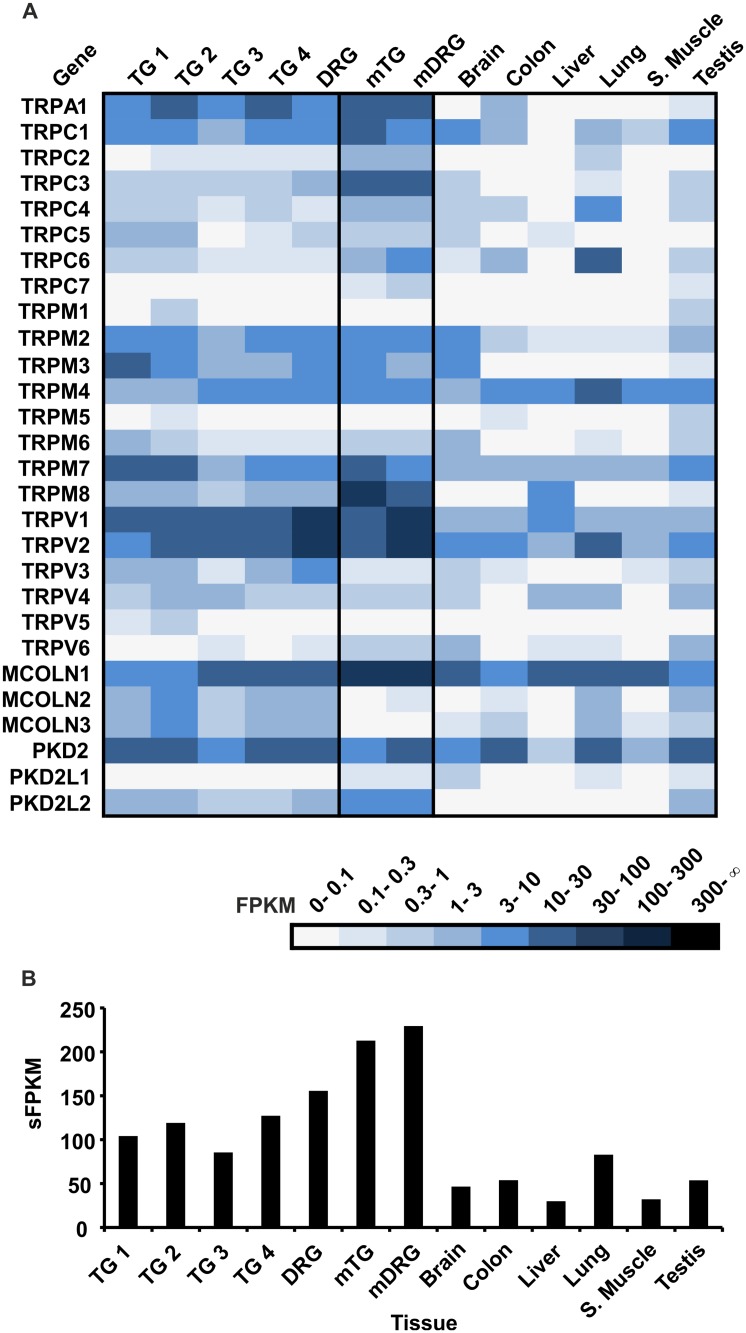
Expression of TRPs in the human TG and DRG. **A** TRP transcripts were detected in the human TG and DRG. **B** Investigation of the sFPKM values of the TRP transcripts across all tissues. The expression of TRP is higher in human sensory ganglia than in the human reference tissues (brain, colon, liver, lung, s. muscle, and testis) but lower than that in the mTG and mDRG.

Our RNA-Seq analysis of the human TG and DRG reveals the tissue selective expression of all TRP channel genes in comparison with non-chemosensory tissues (Fig 6). Here, we found moderate TRPV1 and TRPV2 expression (~10–30 FPKM), which corresponds to data obtained for the mTG (Fig 6A). TRPA1 was also expressed at moderate levels in human TG samples (~3–30 FPKM), which were lower than that in the mTG (Fig 6A). Most strikingly, TRPM8 expression was weak to moderate in our human TG and DRG samples (~0.5–2.5 FPKM), whereas it showed strong expression in the mTG and mDRG (Fig 6A). According to this observation, TRPM8 might play a less prominent role in human than mouse somatosensation. In contrast, TRPV3 expression was moderate in the human TG and DRG but weak in the corresponding mouse ganglia (Fig 6A). In mouse, the warmth sensitive TRPV3 channel is highly expressed in keratinocytes [99]. Upon TRPV3-mediated heat activation, keratinocytes release ATP onto sensory neurons, that in turn, are insensitive to direct activation by heat [118]. According to our expression profiling and immunohistochemistry data [115], TRPV3 might actually be a warmth sensor in human sensory fibers. The thermosensitive TRPV4 channel is a swell-activated osmolarity sensor [97,98,119]. TRPV4 immunoreactivity was shown in human nerve fibers [120,115] and human skin [120,121]. Our RNA-Seq analysis revealed weak TRPV4 expression in all four human TG samples and the DRG with FPKM values comparable to that of TRPV3 (Fig 6A). Similar levels of TRPV4 expression were detected in the mTG and mDRG and human brain, liver, lung, and testis. Furthermore, we found moderate to strong expression of TRPC1, TRPM2, TRPM3, TRPM7, and TRPML1 in the human TG and DRG (Fig 6A). The high expression levels of TRPC1, TRPM7, and TRPML1 were not specific to the TG and DRG but were also found in several non-somatosensory tissues (Fig 6A). Among the non-thermo TRPs, TRPC3 showed weak expression in the human TG and DRG, whereas it was expressed at moderate levels in the corresponding mouse tissues (Fig 6A). We found weak expression of the TRPP channel family member PKD2L1 in human TG and DRG samples and human testis (Fig 6A). This gene is expressed at comparable levels in the mDRG and mTG [34]. In mouse taste buds, PKD2L1 heterodimerizes with PKD1L3 giving rise to acid-sensing channels [122], and its role in somatosensory nerve fibers is unknown.

### Ca^2+^-activated chloride channels

#### ANO

ANO channels are Ca^2+^-activated Cl^-^ channels integral to olfactory signaling in rodents [123,124] and somatosensation [125]. We recently showed that Ca^2+^-activated Cl^-^ currents contribute to signal amplification in murine TG neurons upon the detection of chemical cues [92,3].

**Fig 7:**
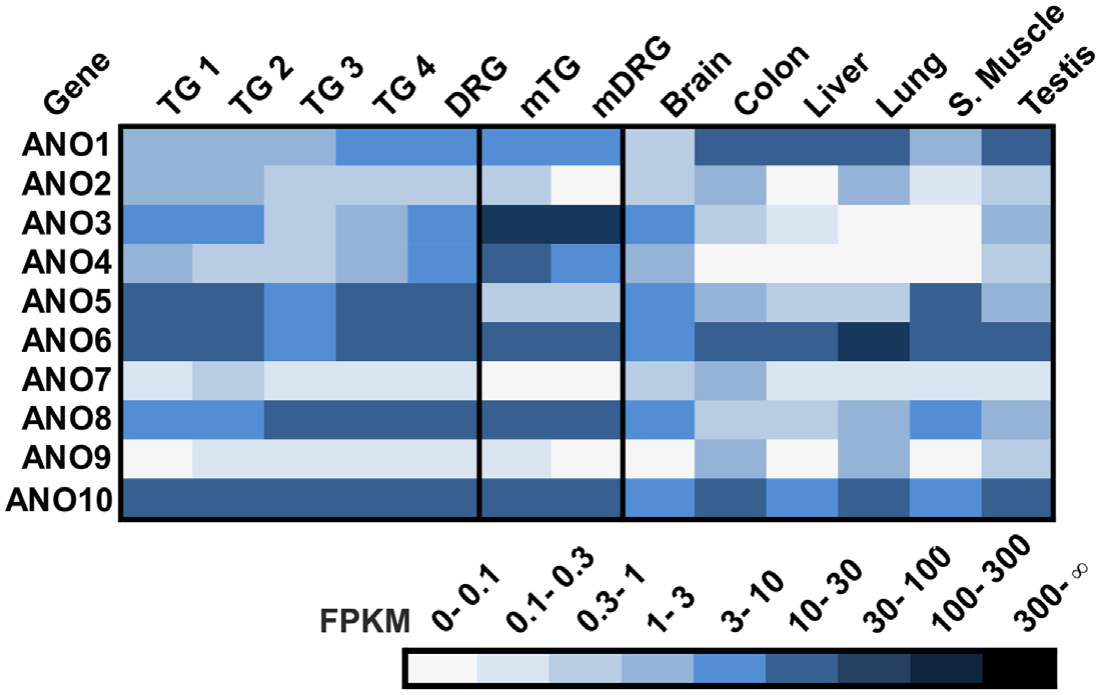
Expression of ANOs in the human TG and DRG. Transcription of ANO channel members was detected in the TG and DRG.

To date, no expression data or immunohistochemical analysis of ANO type Ca^2+^-activated Cl^-^ channels in the human TG and DRG is available. Here, we analyzed the expression of ANO1-10 in human somatosensory ganglia (Fig 7). In general, ANO transcript levels were comparable between the TG and DRG samples. ANO3 through ANO7 are putatively intracellularly localized channels [126]. Due to their intracellular localization, a role in chemosensory signaling is unlikely. FPKM values for these transcripts ranged from <1 FPKM (ANO7), >1 FPKM (ANO3 and ANO4), and >10 FPKM (ANO5 and ANO6) (Fig 7). In RNA-Seq analysis of mTG tissue, Ano3, Ano4, and Ano6 showed the highest FPKM values [92]. ANO1, ANO2, ANO8, and ANO10 give rise to transmembrane Ca^2+^-activated Cl^-^ currents [127], indicating a possible role in fast signaling events in somatosensory neurons. ANO1 is involved in the bradykinin-mediated depolarization of DRG neurons [128] and acts as a heat sensor in these cells [125]. In the mTG, Ano1 is expressed at low levels [92]. In the human TG and DRG, ANO1 and ANO2 have FPKM values >1, corresponding to weak to moderate expression levels (Fig 7). ANO8 and ANO10 show moderate to high expression in the human TG and DRG. Interestingly, the high FPKM values for ANO8 are specific to the sensory ganglia, whereas the high FPKM values for ANO10 were also detected in the colon, lung, and testis (Fig 7). FPKM values for ANO8 and ANO10 are comparable to that of the mTG and mDRG [92,88], indicating that both Ca^2+^-activated Cl^-^ channels have the same physiological functions in these sensory ganglia.

#### TTYH

**Fig 8:**
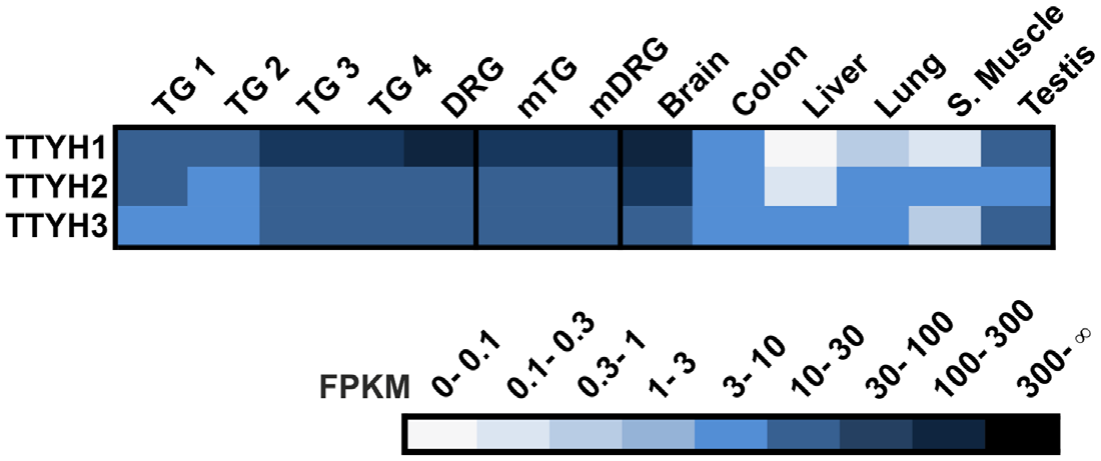
Expression of TTYHs in the human TG and DRG. The transcription of all TTYH channel members was detected in the TG and DRG. The expression of the TTYH genes is higher in the human sensory ganglia than the human reference tissues with the exception of the brain and testis.

Tweety channels (TTYH) form another group of Ca^2+^-activated Cl^-^ channels. We found expression of all three TTYH channels in the human TG and DRG (≤117 FPKM; Fig 8). The expression intensities in the human TG and DRG are relatively high and comparable to that of the mTG and mDRG as well as that of the human brain and testis. To date, the functions of the TTYH channels in human sensory neurons remain unknown.

#### ACCN

**Fig 9:**
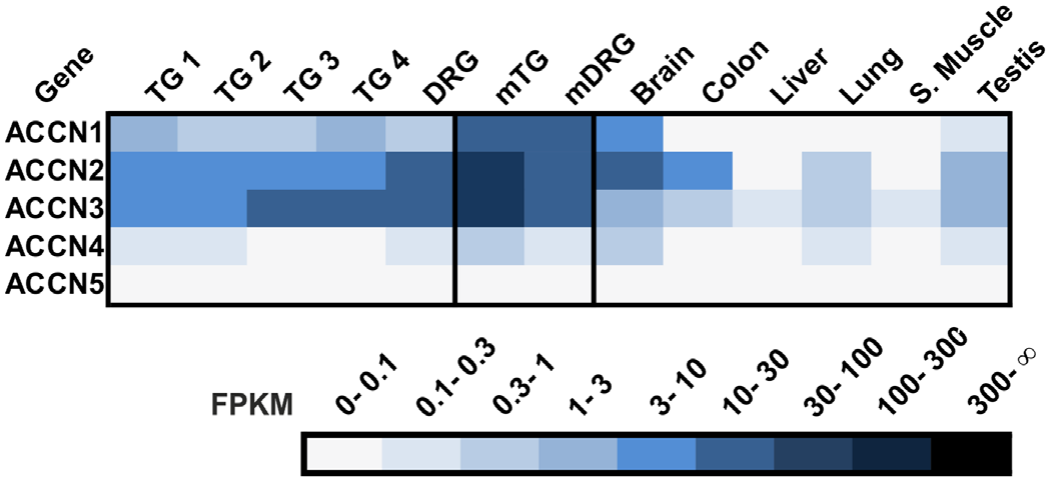
Expression of ACCN in the human TG and DRG. ACCNs 1–3 are highly expressed in sensory ganglia.

Amiloride-sensitive cation channels (ACCN), also known as acid-sensing ion channels (ASICs), are voltage-insensitive, proton-gated Na^+^ channels belonging to a superfamily including the epithelial Na^+^ channel. ACCN subunits possess two putative TM domains and are assembled as hetero- or homotrimers [129,130]. ACCN proteins 1–3 were previously detected in a sub-population of small-diameter sensory neurons in the human DRG [131]. Our datasets confirm that these three receptors are highly expressed in the human TG and DRG with FPKM values ≤27 (Fig 9). ACCN3 is known to be involved in multimodal sensory perception in the sensory ganglia, including nociception, mechanosensation, and chemosensation [132]. ACCN4 and 5 are not or are only weakly expressed in the human TG and DRG (Fig 9).

### Piezo receptors

**Fig 10:**
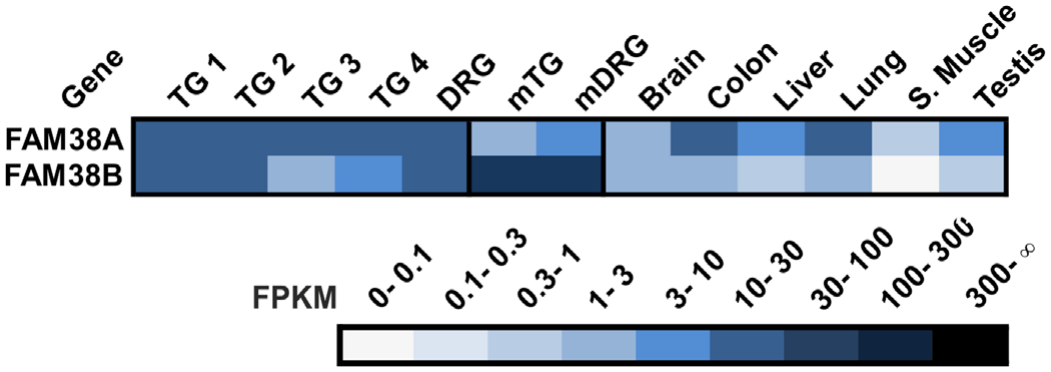
Expression of piezo receptors in the human TG and DRG. FAM38A and B are highly expressed in both sensory ganglia.

FAM38A and B, also known as Piezo1 and 2, are mechanically activated cation channels and may be involved in sensations such as touch and pain [133]. Their expression has not been previously described in the human TG and DRG. FAM38A and B are highly expressed in the TG and DRG with FPKM values ≤17 (Fig 10).

### Purinergic receptors

P2X receptors are involved in a wide range of pathophysiological pain mechanisms, such as inflammatory, neuropathic, acute, and migraine-induced pain [134]. These receptors can define chemosensory properties in rat trigeminal neurons and may contribute to odorant perception via the trigeminal system [10]. Furthermore, we previously showed that keratinocytes act as chemosensors linking the environment and the trigeminal system via ATP signaling [135].

**Fig 11:**
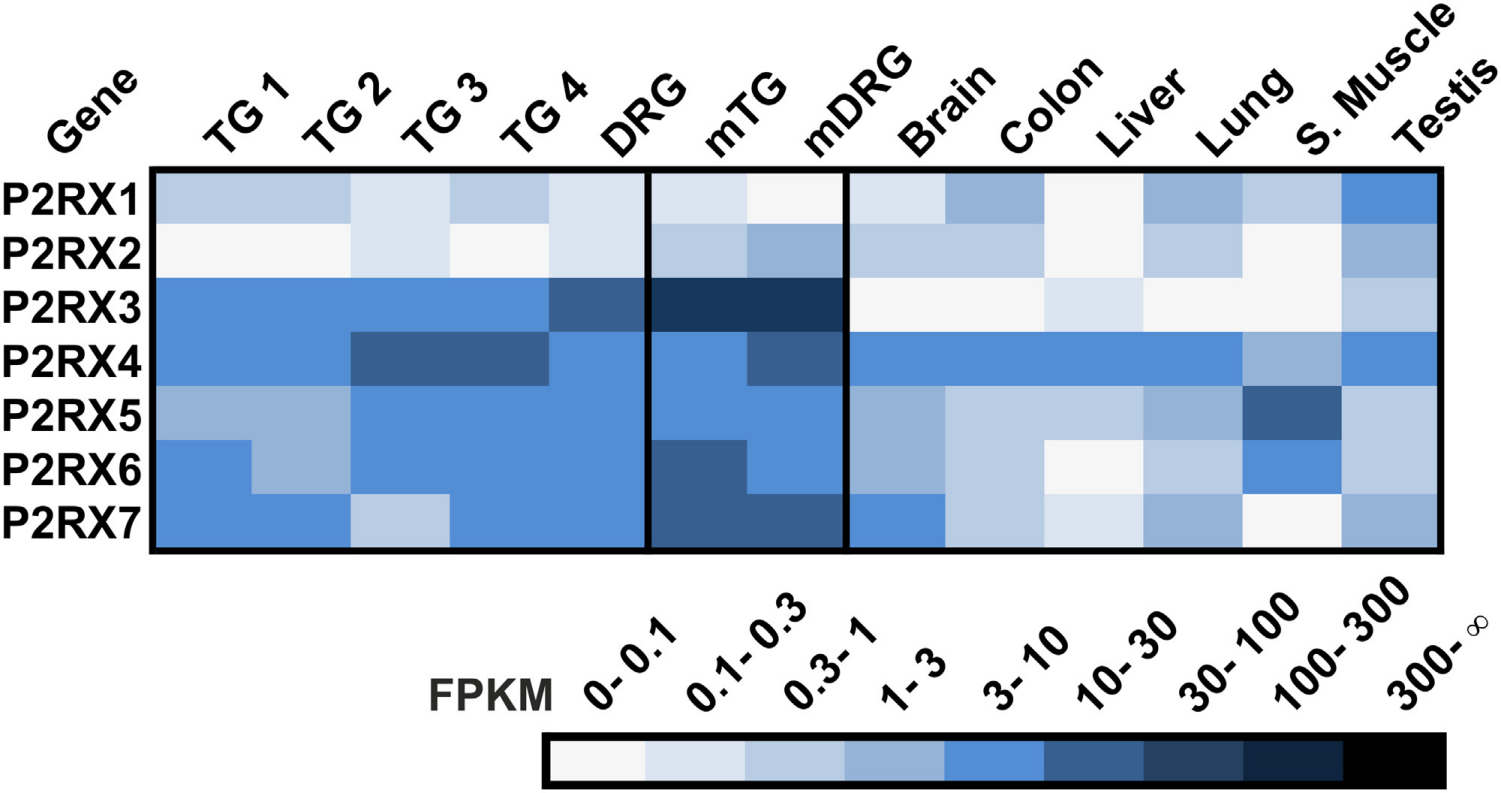
Expression of P2XRs in the human TG and DRG. The ionotropic purinergic receptors are highly expressed in sensory ganglia. Five of the seven P2XRs are expressed higher in the TG and DRG than in any other tissue.

We identified the expression of the ionotropic purinergic receptor class P2X in the human TG and DRG (Fig 11). The expression of P2RX2-7 has been well described in the sensory ganglia of rodents but not in humans. Our RNA-Seq results revealed that P2RX3 is highly expressed in sensory ganglia, whereas it is almost absent in reference tissues investigated (Fig 11). The expression of P2RX3 has been described for human fetal DRG, where it is generally localized in small-sized neurons (<20 μm diameter) [136]. Moreover, the high P2RX3 expression is consistent with its expression in mTG and mDRG and for all of the additionally expressed P2RXs [34] (Fig 11). P2RX1 and P2RX4-7 are broadly and moderately expressed in almost all human tissues investigated, but they are highly expressed in sensory ganglia (Fig 11).

### Potassium channels

In addition to TRP channels, potassium channels are involved in somatosensation and nociception in sensory nerves [137,138]. Potassium channels are subclassified as voltage-gated (KCNA-KCND, KCNF-KCNH, KCNQ and KCNS), calcium-activated (KCNM-KCNN), inward rectifying (KCNJ), and background/leak, 2 pore (KCNK) channels [139]. Expression analysis of all potassium channels can be found in S11 Fig.

### KCNK

The 15 KCNK channel members are a class of thermo-, mechano-, and chemosensitive membrane proteins that are involved in nociception and somatosensation [137]. KCNKs 3, 9, and 18 function as chemoreceptors for hydroxyl α-sanshool in murine TG neurons [90]. Additionally, these three human KCNKs are inhibited by the pungent substances piperine, capsaicin, 6-gingerol, and polygodial [91]. Furthermore, human KCNKs 2, 10, and 18 are blocked by high concentrations of (-)-nicotine [140]. Expression in human DRG was already described for KCNK1-7, 10 and, 18 [141,142].

**Fig 12:**
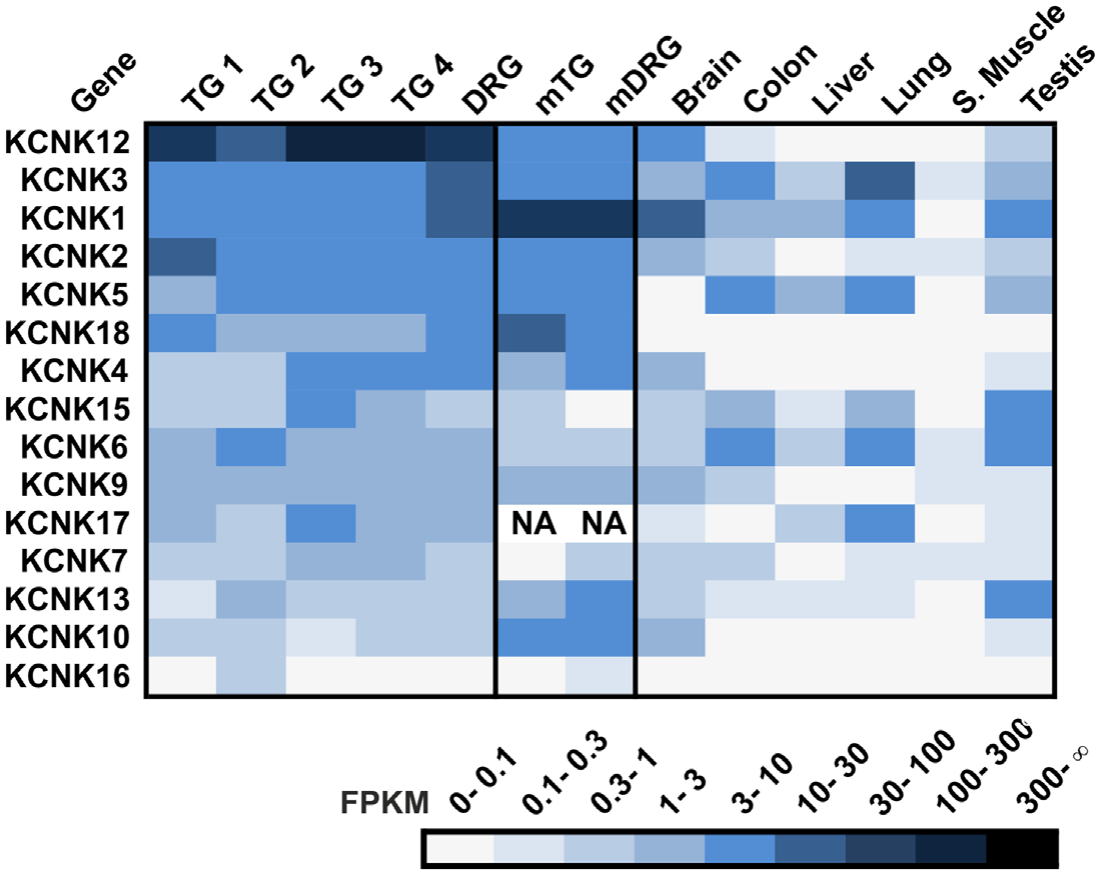
Expression of KCNK channels in the human TG and DRG. All KCNK genes are expressed in the TG and DRG. The KCNK genes are sorted by the mean of their expression values across all human sensory ganglia.

Our RNA-Seq data allowed for the complete expression analysis of human DRG and TG and revealed the expression of virtually all human annotated KCNK channels in all sensory ganglia investigated (Fig 12). As an exception, KCNK16 was detected only in one TG sample (TG 2). We detected the highest mean transcript levels (mean FPKM) in the TG and DRG for KCNK12 (mean FPKM: 62.3), KCNK3 (mean FPKM: 7.1), and KCNK1 (mean FPKM: 6.5). For KCNK12, which was most highly and specifically expressed, the function is still unknown [143]. The three most highly expressed KCNK genes in murine sensory ganglia differed in the human expression results (Kcnk1, Kcnk18, and Kcnk2). Our data indicate the exclusive expression for KCNK18 in the human TG and DRG (mean FPKM: 2.8) (Fig 12), which was also detected with RT-PCR experiments by Lafrenière and colleagues [142]. Murine RNA-Seq data also indicate selective KCNK18 expression in the mTG and mDRG [34]. A mutation within the KCNK18 open reading frame leads to a non-functional protein and has been found to be associated with migraines [142].

### Other GPCRs and ion channels

**Fig 13:**
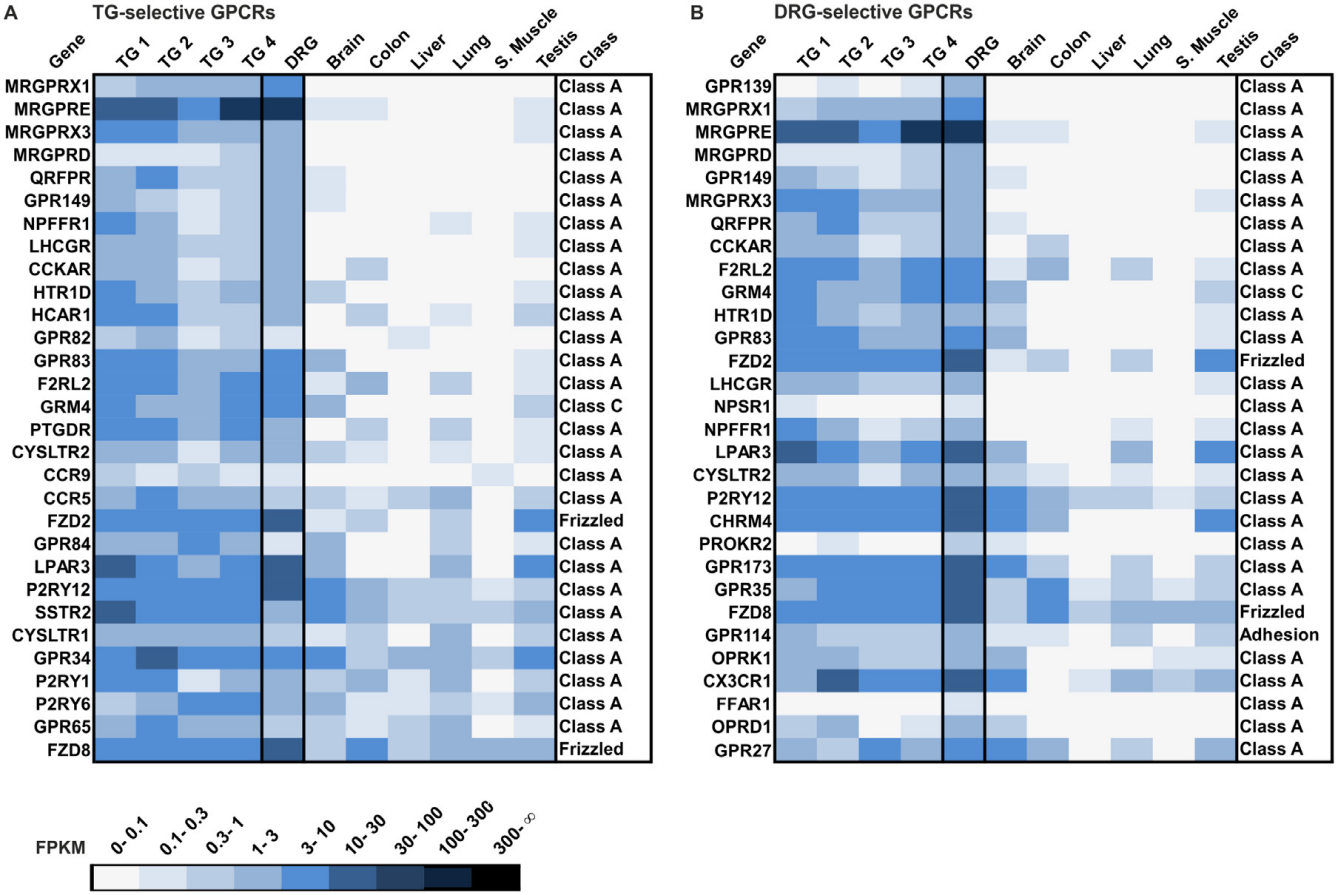
Expression analysis of the 30 most selectively expressed GPCRs in the human TG and DRG. **A** The heatmap shows the 30 most selectively expressed GPCR transcripts in the TG in comparison with the DRG and reference tissues (brain, colon, liver, lung, s. muscle, and testis). Genes are ranked according to their selective expression in the TG (Quotient of the mean FPKM value (TG1-4) and the mean FPKM value for all reference tissues; only the transcripts detected in all four TG samples with FPKM >0.1 are taken into account). MRGPRs are the most selective GPCRs that have been detected. **B** Shown are the 30 most selectively expressed GPCRs in the DRG. Genes are ranked according to their selective expression in DRG. The ranking was calculated by the quotient of the FPKM value of DRG (FPKM >0.1) and the mean FPKM value for all reference tissues. GPR139 is the most specific GPCR followed by three MRGPRs, which have been selectively detected in the human DRG.

Furthermore, we analyzed the transcript levels of all annotated non-olfactory GPCRs. A list of non-olfactory GPCR genes (375 different genes) was generated based on the IUPHAR database. In total, we detected 273 and 260 GPCRs expressed in the human TG (mean number of TG 1–4) and DRG (FPKM > 0.1). We generated a ranking of the 30 most selectively expressed GPCRs in the human TG and DRG (Fig 13). Among the 30 most selectively expressed GPCR transcripts in the human TG and DRG, we detected GPCRs known to be involved in chemoreception (e.g., MRGPRX1 [19]), nociception (e.g., MRGPRD [86]), migraines (e.g., HTR1D [144]), and inflammation (e.g., F2RL2 [145]). The three most selectively expressed GPCRs in the human TG are MRGPRX1 (mean FPKM: 1.8), MRGPRE (mean FPKM: 17) and MRGPRX3 (mean FPKM: 3.4). In the DRG, we detected GPR139 (FPKM: 1.1), MRGPRX1 (FPKM: 8.7) and MRGPRE (FPKM: 66.9) as the three most selectively expressed GPCRs. The human TG and DRG share 18 of the 30 most highly expressed GPCRs.

**Fig 14:**
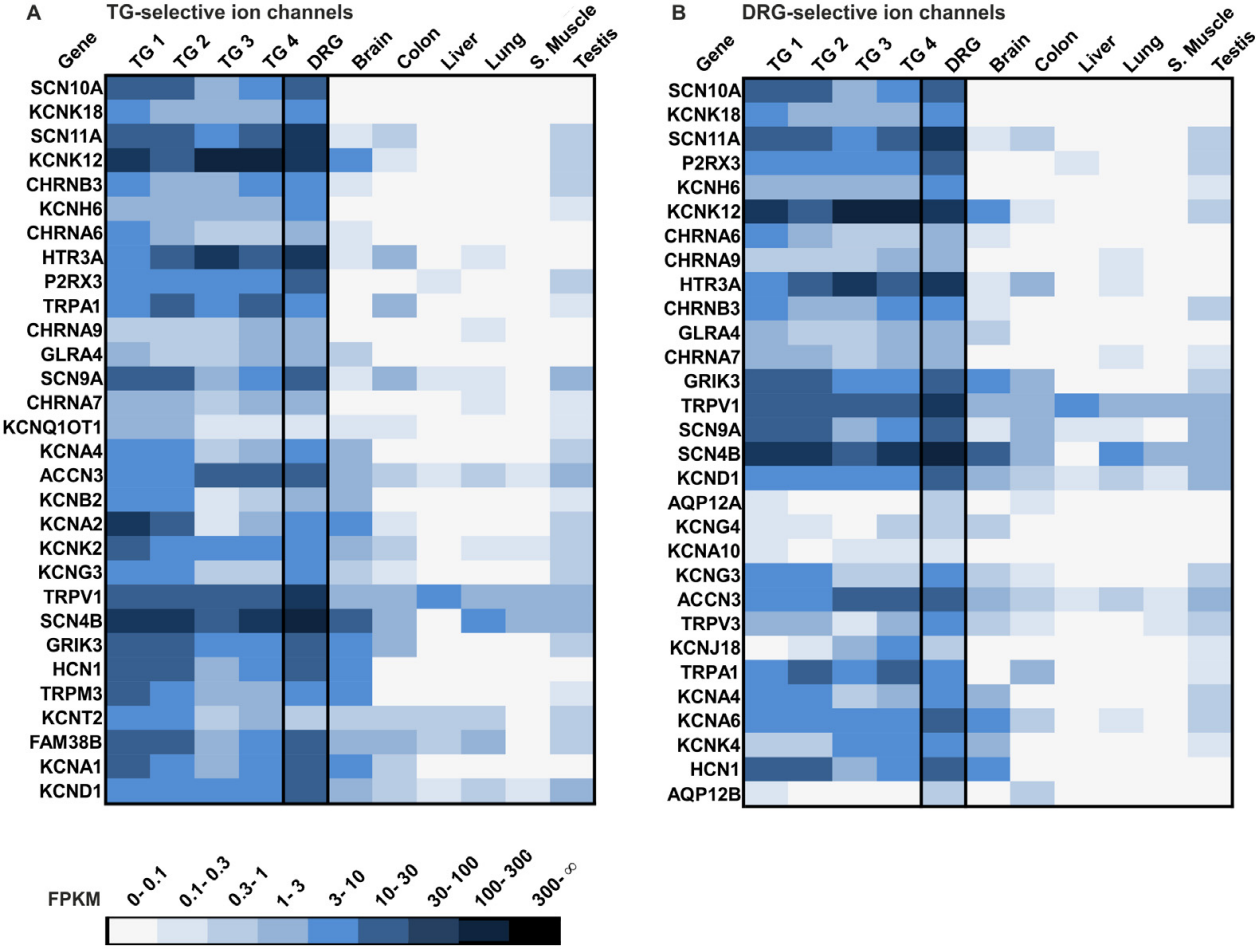
Expression analysis of the 30 most selectively expressed ion channels in the human TG and DRG. A The heatmap shows the 30 most selectively expressed ion channels in the TG compared with the DRG and reference tissues (brain, colon, liver, lung, s.muscle, and testis). Genes are ranked according to their selective expression in the TG (Quotient of the mean FPKM value (TG1-4) and the mean FPKM value of all reference tissues, only those genes expressed in all four TG with FPKM >0.1 were taken into account), and of which SCN10A is the most selectively expressed ion channel in the human TG. B Shown are the 30 most selectively expressed ion channels in the DRG. The genes are ranked according to their selective expression in the DRG. The ranking was calculated by the quotient of the FPKM value in the DRG (FPKM >0.1) and the mean FPKM value for all reference tissues. SCN10A is the most selective ion channel detected in the DRG.

Next, we investigated the expression of the most common ion channels in the human TG and DRG (351 different genes) based on [146]. In total, we found 304 ion channels in the TG and 299 in the DRG to be expressed (0.1> FPKM). Fig 14 shows the 30 most selectively expressed ion channels in the human TG and DRG. The expression pattern of the selectively expressed ion channel genes is similar between the TG and DRG, and 21 ion channels are the most selectively expressed ion channels in both tissues. SCN10A, KCNK18, and SCN11A are the three most selective ion channel genes in both, the TG and DRG. More than half of the 30 most selectively expressed ion channels in the human TG and DRG play a role in diverse sensory functions. Among these channels are the previously described KCNK, TRP, P2RX, and ACCN channels and the FAM38 receptors. SCN10A and 11A, which belong to the family of voltage-gated sodium channel α-subunits SCNA were found to be highly selectively expressed in the human TG and DRG. These channels are known to be involved in orofacial or trigeminal neuropathic pain and toothache [147–150]. In the TG and DRG, we detected the same four members of the nicotinic acetylcholine receptors (CHRNA6, 7, 9, and B3), which possibly act as chemosensors for (-)-nicotine [140]. Ionotropic kainate glutamate receptor 3 (GRIK3) is selectively expressed in the human TG and DRG. In insects, this receptor is associated with chemoreception [151]. In humans, polymorphisms within this gene are associated with several diseases such as schizophrenia [152].

### Genes associated with olfactory signaling

**Fig 15:**
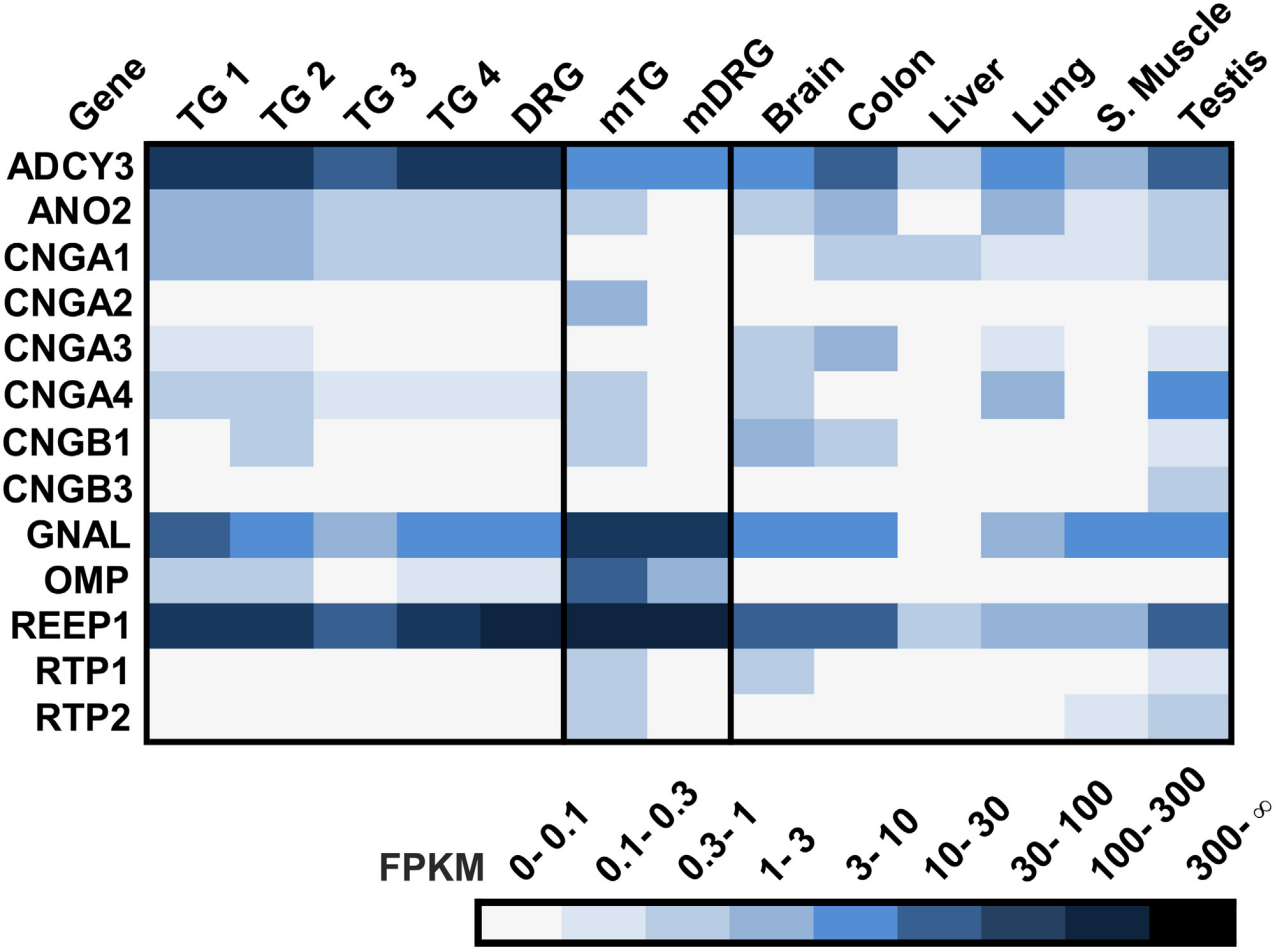
Expression of olfactory signal transduction components in the human TG and DRG. Expression pattern of signal transduction components including adenylyl cyclase III (ADCY3), the calcium-activated chloride channel (ANO2), the classical olfactory and rod CNG channel subunits (CNGA1, CNGA2, CNG3, CNGA4, CNGB1 and CNGB3), and Gα_olf_ (GNAL). We also investigated the expression of accessory proteins including receptor-transporting proteins (RTP1 and RTP2) and receptor-enhancing proteins 1 (REEP1) as well as the expression of olfactory marker protein (OMP), a specific marker for olfactory sensory neurons.

Next, we determined the expression pattern of the signaling components of a potential olfactory signal transduction cascade. In olfactory neurons, ORs couple to a cAMP-mediated second messenger cascade that involves Gα_olf_, adenylyl cyclase III, and the subunits of the CNG channel CNGA2, CNGA4 and CNGB1 [153]. Two of the essential components of olfactory signal transduction, the Gα subunit Gα_olf_ and adenylyl cyclase III (ADCY3), are highly expressed in the human sensory ganglia, but no pronounced expression of the corresponding olfactory CNG channel was detected (Fig 15). Lately, the olfactory CNG channel CNGA2 was identified in mTG tissue by RNA-Seq [34] indicating the presence of a cAMP target channel in mTG neurons. In human sensory ganglia, ORs could couple to G_olf_ and increase cAMP by ADCY3 activation; however, cAMP does not trigger Ca^2+^ influx similar to olfactory sensory neurons by opening olfactory CNG channels. In other tissues, ectopically expressed ORs use different signaling mechanisms. In keratinocytes OR activation leads to the opening of the rod CNG channel [59], and TRPV6 is involved in the OR signal transduction cascade in prostate cancer cells [57].

## Conclusions

Somatosensation by the TG and DRG is an important characteristic of human sensory physiology that is relevant to nutrition, the enjoyment of food, peripheral mechanisms, such as itch and pain, the regulation of temperature, physiology of the skin, environmental toxicology, eye pathologies, and respiratory diseases. Our results provide a framework for insight into the molecular basis of these different sensations and processes. We detected and described a broad panel of different chemoreceptors, and the receptor expression patterns align with the known functions of the TG and DRG. The expression of some receptors, such as ORs, was described here for the first time. In addition to testis tissue, the TG could be defined as a tissue with the highest number of expressed OR genes.

## Materials and Methods

### Ethics Statement

Human trigeminal ganglia samples were obtained from the archives of the institution of pathology of the Ruhr-University Bochum following appropriate institutional approval by the ethical committee of the medical faculty of the Ruhr-University Bochum (file number 5145–14). The ethical committee waived the need for written consent. The samples were received and analyzed anonymously. The committee did not raise any objections against this study.

### Human tissues and RNA isolation

A total RNA pool from 21 different human DRG was purchased from a commercial source (Clontech, CA, USA). Human trigeminal ganglia samples were obtained at autopsy from male adult subjects at the institution of pathology of the Ruhr-University Bochum. The isolation of RNA was performed using the RNeasy Mini Kit (Qiagen, Hilden, Germany) according to the manufacturer’s protocol. The RNA samples were subjected to DNaseI treatment using the TURBO DNA-free Kit (Life Technologies, Carlsbad, CA, USA) according to the standard protocol.

### RNA-Seq by next generation sequencing

At the Cologne Center for Genomics NGS unit, libraries for sequencing were constructed from total or mRNA. Libraries from total RNA were subjected to DSN normalization. RNA-Seq was performed on the Illumina GAIIx (read length: 36 bp single and paired-end) or HiSeq 2000 sequencing platforms (101 bp paired-end). The Body Map 2.0 data used in this study as a reference were obtained as previously described [41].

### Alignment of RNA-Seq reads using TopHat

We analyzed the RNA-Seq data as previously described [41]. RNA-Seq reads were aligned to the hg19 reference genome by TopHat v1.2.0. Aligned data were visualized with the Integrative Genomic Viewer [63]. The command line used for TopHat was as follows:

tophat—output-dir <name output>—GTF <hg19refseq.gtf> <indexes> tissue.fq

### Alignment assembly and gene expression using Cufflinks

Cufflinks v1.0.3 software was used to calculate the abundance of transcripts based on the RefSeq gene model as described in [41]. The relative abundance of transcripts was reported in FPKM (fragments per kilobase of exon per million fragments mapped) units [154]. The Cufflinks parameters are listed below.

cufflinks—output-dir <name output2>—GTF <hg19refseq.gtf>—multi-read-correct—compatible-hits-norm—minfrags-per-transfrag 1—frags-bias-correct <hg19.fa> sorted.bam

### cDNA synthesis and semi quantitative RT-PCR

cDNA synthesis was performed according to the previously described protocol of [41]. An equivalent of 50 ng of total RNA was used for each RT-PCR experiment. To validate the expression of different ORs, we designed primers that detect ~100–300 bp fragments of OR ORFs. PCR was performed using GoTaq qPCR Master Mix (Promega, Madison, WI, USA) with the Mastercycler RealPlex^2^ (Eppendorf, Hamburg, Germany) (20 μl total volume, 40 cycles of 95°C, 59°C, and 72°C at 45 s each). All experiments were conducted in triplicate. For a semi-quantitative analysis we normalized probes to 3 ng of genomic DNA. A list of primers used can be found in S12 Fig.

### Microarray analysis

Gene expression analysis of trigeminal ganglia (n = 3) and reference tissues (liver, heart, muscle, skin) was performed using a customized Agilent Whole Human Genome Microarray 8x15k from Miltenyi Biotech GmbH (Bergisch Gladbach, Germany). The microarray contains triplicate probes 50 nt in length for ~850 OR genes or pseudogenes. Total RNA was isolated as described above. The quality of the total RNA was controlled for using the Agilent 2100 Bioanalyzer System. Linear amplification of the RNA and hybridization reactions as performed according to Agilent’s standard protocols. Pre-analysis of the data, including median normalization of the intensity values, was performed by the Miltenyi Biotec GmbH using Agilent Feature Extraction Software. For the expression analysis of ORs in TG 1, normalized mean intensities of three oligonucleotides per array were normalized to different reference tissues.

### Cell culture and transfection

Hana3A cells were used for recombinant OR expression to test specificity of the OR antibodies. The Hana3A cells were kindly provided by H. Matsunami (Duke University Medical Center, Durham, NC). The Hana3A cells were maintained under standard conditions as previously described [76] and grown on cover slips in 24-well plates. Cells were transfected with Lipofectamine 2000 (Invitrogen, Carlsbad, CA, USA) according to the manufacturer’s protocol with 300 ng of OR plasmid and 60 ng of mRTP1S plasmid (constructed using standard PCR methods).

For the antibody specificity studies, the OR6B2 coding sequence was PCR amplified from human genomic DNA using specific primer pairs (for: 5’-GCATATGAATTCATGAGTGGGGAGAATGTCACC-3’ and rev: 5’-GCATATGCGGCCGCTCAGTGTGAAGTTTGACCCAAGC-3’) that amplify the complete open reading frame and contain the EcoRI and NotI restriction sites for further subcloning into the pCI plasmid (Promega, Madison, USA), which contain the coding sequence for the N-terminal rhodopsin tag (rho-tag, first 20 amino acids of rhodopsin). All plasmid constructs and PCR products were verified by Sanger sequencing.

### Immunocytochemical and—histochemical staining

Transfected Hana3A cells grown on cover slips were fixed with 4% paraformaldehyde. Commercially available human DRG paraffin tissue sections with a thickness of 5 μm (Zyagen, San Diego, CA, USA) were deparaffinized by dipping slides in xylene (2 changes, 5 min each), rehydration in a serial ethanol solutions, and finally washing in PBS^-^/^-^. For the deparaffinized slides, antigen retrieval with citrate buffer was performed (20 min, 90°C). Afterward, fixed cells and deparaffinized tissue slides were permeabilized with PBS^-^/^-^ + 0.1% Triton X-100, washed with PBS^-^/^-^, and incubated with blocking reagent (PBS^-^/^-^ + 0.1% Triton X-100, 5% NGS, and 1% fish gelatine) for 1 h. Cells and tissue slides were incubated overnight (4°C) with primary antibody and then incubated with the indicated Alexa-conjugated secondary antibodies (Invitrogen, Carlsbad, USA) and DAPI for 45 min at room temperature in the dark. The following antibodies were used: anti-OR6B2 (polyclonal, 1:30, C-terminal, NBP1-71360, Novus Biologicals, Cambridge, UK) and anti-Rhodopsin 4D2 (monoclonal, 1:250, Abcam, Cambridge, UK). The samples were mounted in ProLong Gold (Invitrogen, Carlsbad, USA). Images were obtained using a confocal fluorescent microscope (LSM 500 Meta; Carl Zeiss, Oberkochen, Germany).

## Supporting Information

S1 Fig Expression of housekeeping genes in the human TG, DRG, and reference tissues. Shown are highly expressed genes (ß-actin (ACTB) and glyceraldehyde 3-phosphate dehydrogenase (GAPDH)), moderately expressed genes (ribosomal protein L29 (RPL29) and ribosomal protein L13A (RPL13A)) and weakly expressed genes (β-glucuronidase (GUSB), transferrin receptor (TFRC), hypoxanthine phosphoribosyltransferase 1 (HPRT1) and TATA box binding protein (TBP)).
(TIF)

S2 Fig Distribution of the FPKM values in the human TG. To gain an estimate of the FPKM values for expressed genes, a histogram of FPKM distribution for the TG 3 sample was calculated. Values <0.3 can be regarded as indicating weak expression, 0.3–3 as weakly expressed and 3–30 as moderately expressed. Values of 30–100 indicate high expression, and values >100 indicate extremely high expression. Of the ~23,000 analyzed genes, expression at >0.1 FPKM was detected for ~17,000 genes; mRNA for ~500 of these genes were extremely highly expressed with FPKM values >100.
(TIF)

S3 Fig Number of expressed OR transcripts in the human TG and DRG. Each bar represents the number of OR genes that were expressed in each tissue with an FPKM value >0.1. For the TG, the average number of ORs is shown.
(TIF)

S4 Fig Validation of detected OR splicing events for OR2W3 and OR2L13 by RT-PCR. Detected splicing events could be validated by RT-PCR; and example is shown for DRG. OR2W3: Ex3 (forward primer in exon 3 of TRIM58 and reverse primer in OR2W3 ORF); Ex5 (forward primer in exon 5 of TRIM58 and reverse primer in OR2W3 ORF). OR2L13: Ex1 (forward primer in known exon 1 of 5’UTR and reverse primer in OR2L13 ORF); Ex2 (forward primer in known exon 2 of 5’UTR and reverse primer in OR2L13 ORF). The amplified PCR products were confirmed by Sanger sequencing.
(TIF)

S5 Fig The OR6B2 antibody specifically detects recombinantly expressed OR6B2 in Hana3A cells. Immunostaining of Hana3A cells transiently transfected with OR6B2. The cells were stained with a specific OR6B2 antibody (OR6B2, green) and a rhodopsin-antibody (rho, red). DAPI staining (blue) was used to confirm the number and localization of cell nuclei. Scale bars: 20 μm.
(TIF)

S6 Fig Secondary antibody control in human DRG slides. Control staining was performed without primary antibody, showing non specific staining by the secondary antibody (Alexa Fluor 488 Goat Anti-Rabbit, Control). Scale bar: 20 μm.
(TIF)

S7 Fig Expression of TAARs in the human TG and DRG. No TAAR transcripts could be detected in the sensory ganglia investigated.
(TIF)

S8 Fig Expression of VNRs in the human TG and DRG. Shown are the FPKM values for VNR transcripts in sensory ganglia compared to different reference tissues (brain, colon, liver, lung, s. muscle, and testis).
(TIF)

S9 Fig Expression of taste receptors in the human TG and DRG. The FPKM values for TAS1Rs and TAS2Rs are shown. The sequencing read distribution was checked in the IGV, and in some cases, unclear expression in the human TG and DRG was revealed. Most of the detected TAS2R lay within introns in the moderately expressed PRH1-PRR4 gene. Intronic reads within the PRH1-PRR4 gene could stem from unprocessed transcripts of PRH1-PRR4 and not TAS2R transcripts as described in [155].
(TIF)

S10 Fig The MRGPR transcript in the human TG. A large 3’UTR could be detected in transcripts from human MRGPRs (the example shown is MRGPRE), which is found in mice.
(TIF)

S11 Fig Expression of potassium channels in the human TG and DRG. Genes are sorted by the mean of their expression values across all human sensory ganglia.
(TIF)

S12 Fig Primer sequences used for PCR and splice transcript validation. (TIF)

S1 Table Expression of all genes in the human TG and DRG. Shown are the FPKM values for all expressed genes in sensory ganglia compared to different reference tissues (brain, colon, liver, lung, s. muscle, and testis).
(XLSX)
