# Supplementary figures and images for: RNA-Seq Analysis of Human Trigeminal and Dorsal Root Ganglia with a Focus on Chemoreceptors
Source: PLoS One. 2015 Jun 12;10(6):e0128951. doi: 10.1371/journal.pone.0128951 (PMC4466559; doi:10.1371/journal.pone.0128951)

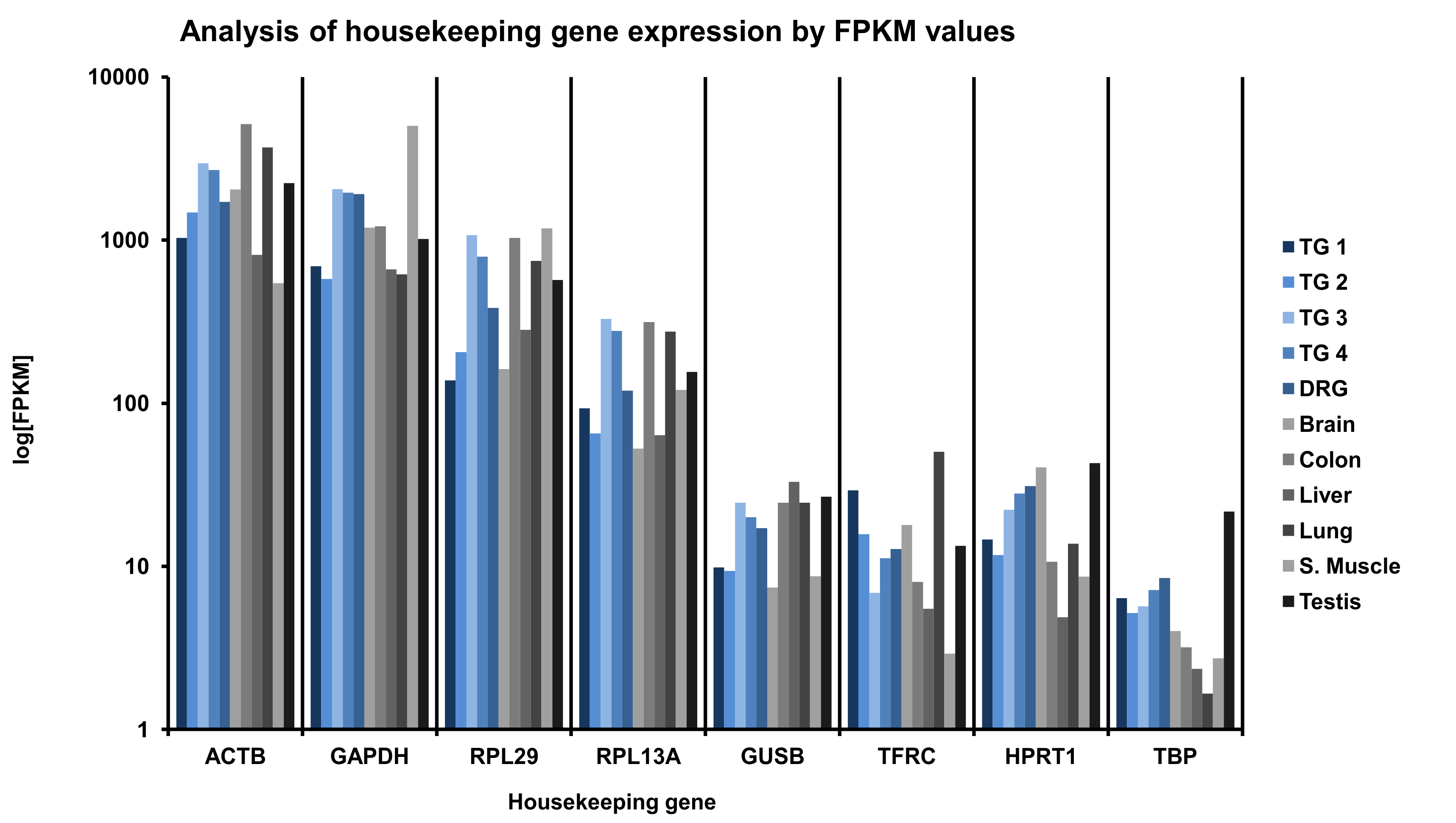

Supplement: S1 Fig — Shown are highly expressed genes (ß-actin (ACTB) and glyceraldehyde 3-phosphate dehydrogenase (GAPDH)), moderately expressed genes (ribosomal protein L29 (RPL29) and ribosomal protein L13A (RPL13A)) and weakly expressed genes (β-glucuronidase (GUSB), transferrin receptor (TFRC), hypoxanthine phosphoribosyltransferase 1 (HPRT1) and TATA box binding protein (TBP)). (TIF) [file pone.0128951.s001.tif]

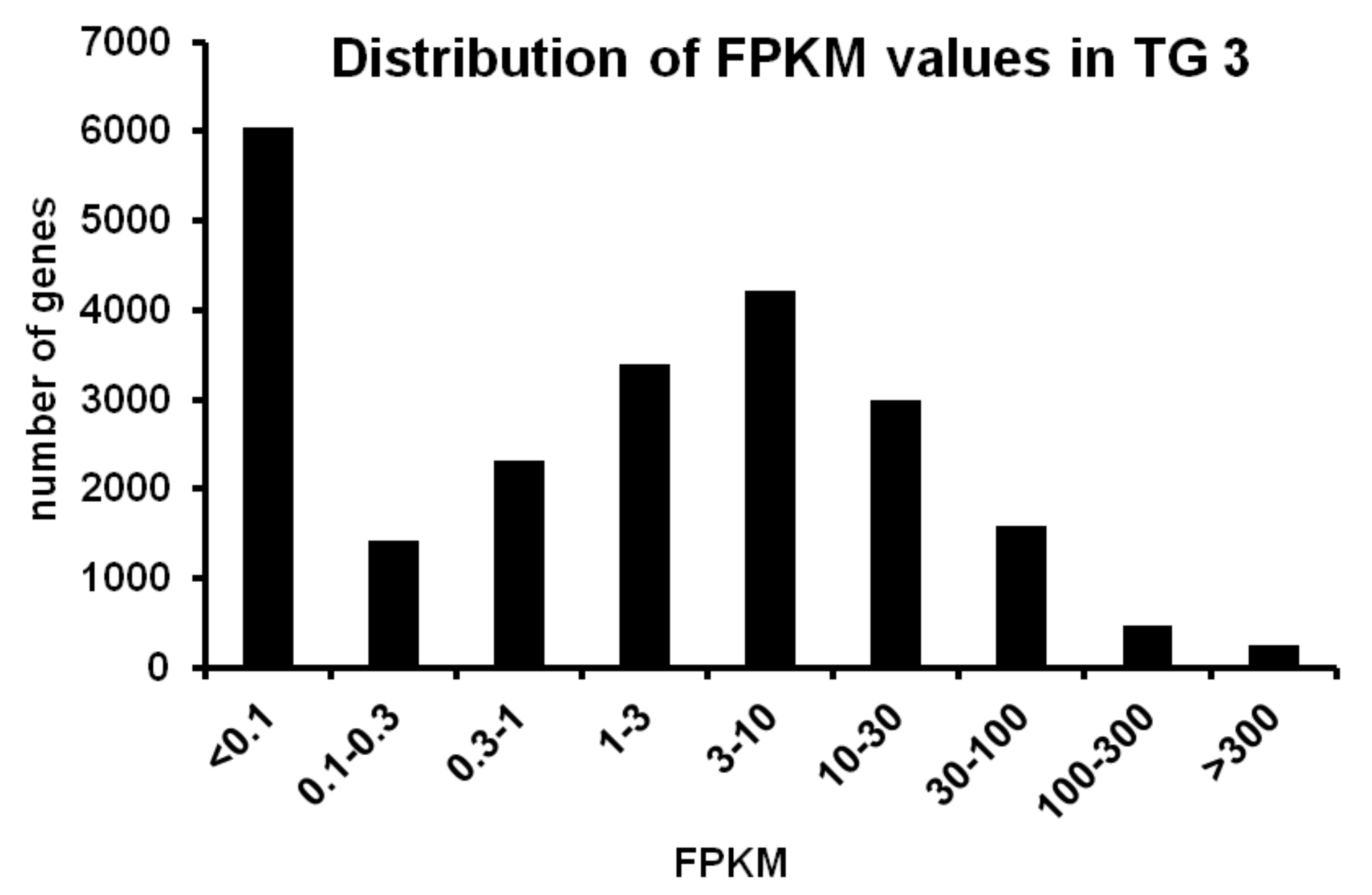

Supplement: S2 Fig — To gain an estimate of the FPKM values for expressed genes, a histogram of FPKM distribution for the TG 3 sample was calculated. Values <0.3 can be regarded as indicating weak expression, 0.3–3 as weakly expressed and 3–30 as moderately expressed. Values of 30–100 indicate high expression, and values >100 indicate extremely high expression. Of the ~23,000 analyzed genes, expression at >0.1 FPKM was detected for ~17,000 genes; mRNA for ~500 of these genes were extremely highly expressed with FPKM values >100. (TIF) [file pone.0128951.s002.tif]

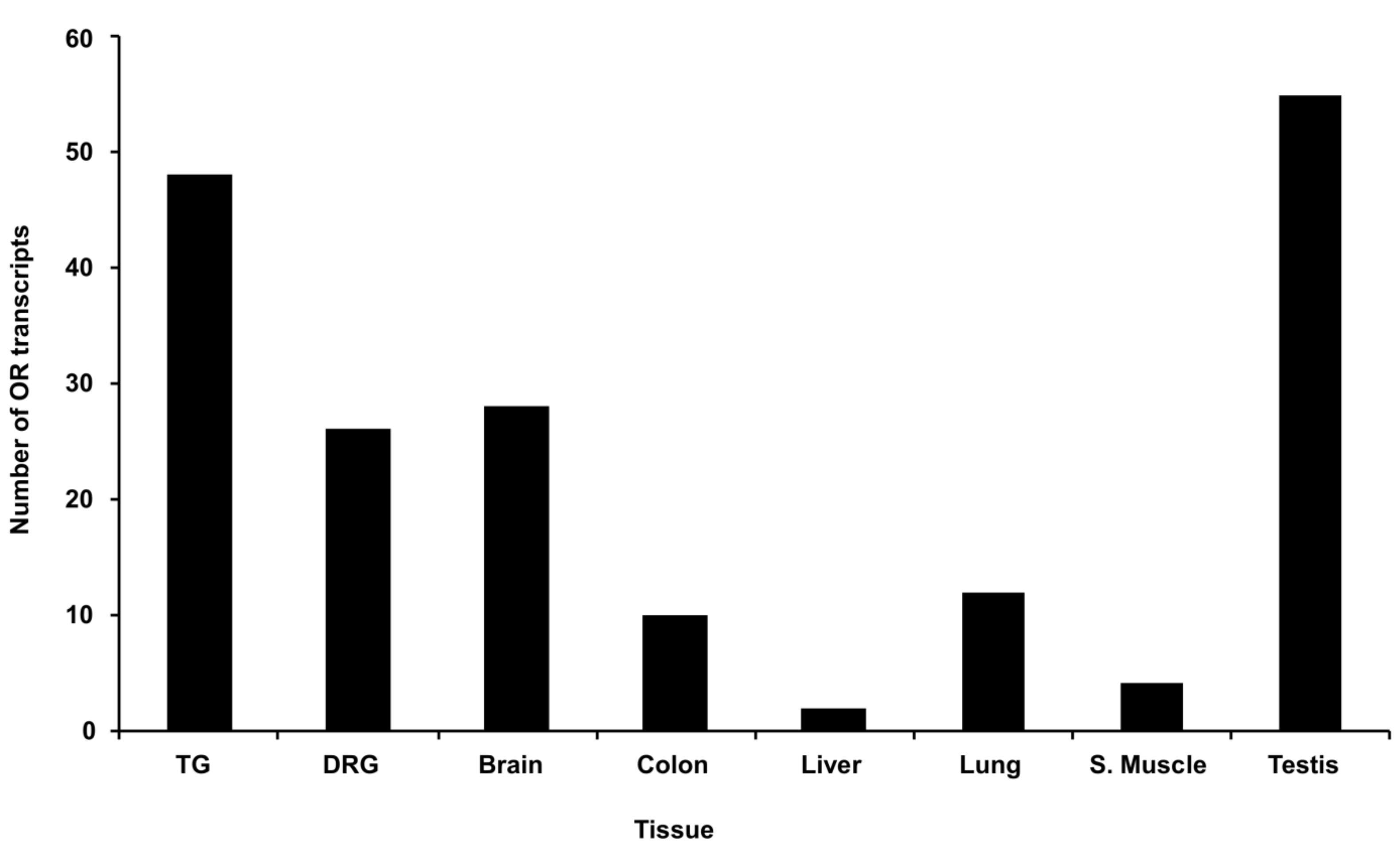

Supplement: S3 Fig — Each bar represents the number of OR genes that were expressed in each tissue with an FPKM value >0.1. For the TG, the average number of ORs is shown. (TIF) [file pone.0128951.s003.tif]

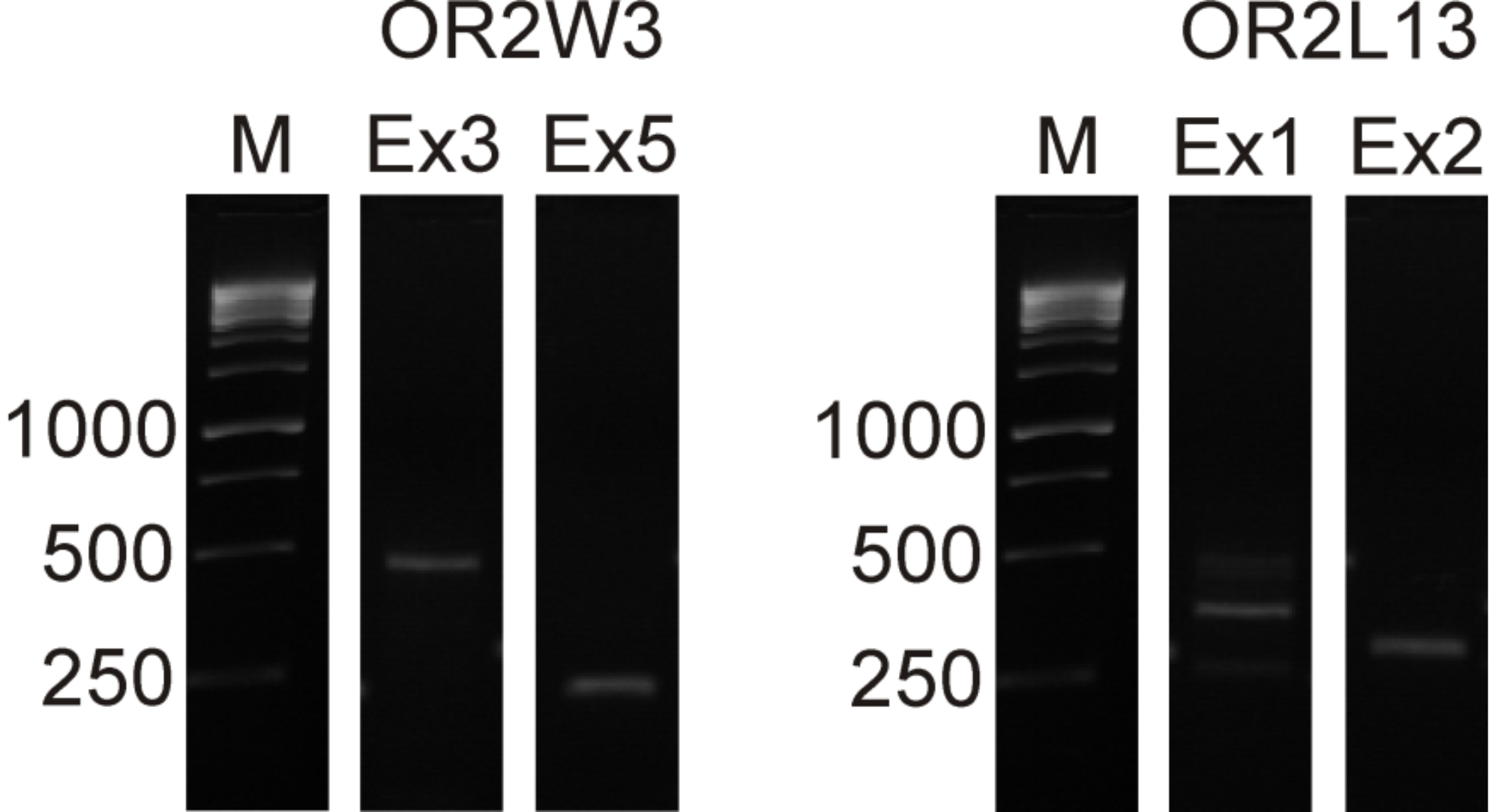

Supplement: S4 Fig — Detected splicing events could be validated by RT-PCR; and example is shown for DRG. OR2W3: Ex3 (forward primer in exon 3 of TRIM58 and reverse primer in OR2W3 ORF); Ex5 (forward primer in exon 5 of TRIM58 and reverse primer in OR2W3 ORF). OR2L13: Ex1 (forward primer in known exon 1 of 5’UTR and reverse primer in OR2L13 ORF); Ex2 (forward primer in known exon 2 of 5’UTR and reverse primer in OR2L13 ORF). The amplified PCR products were confirmed by Sanger sequencing. (TIF) [file pone.0128951.s004.tif]

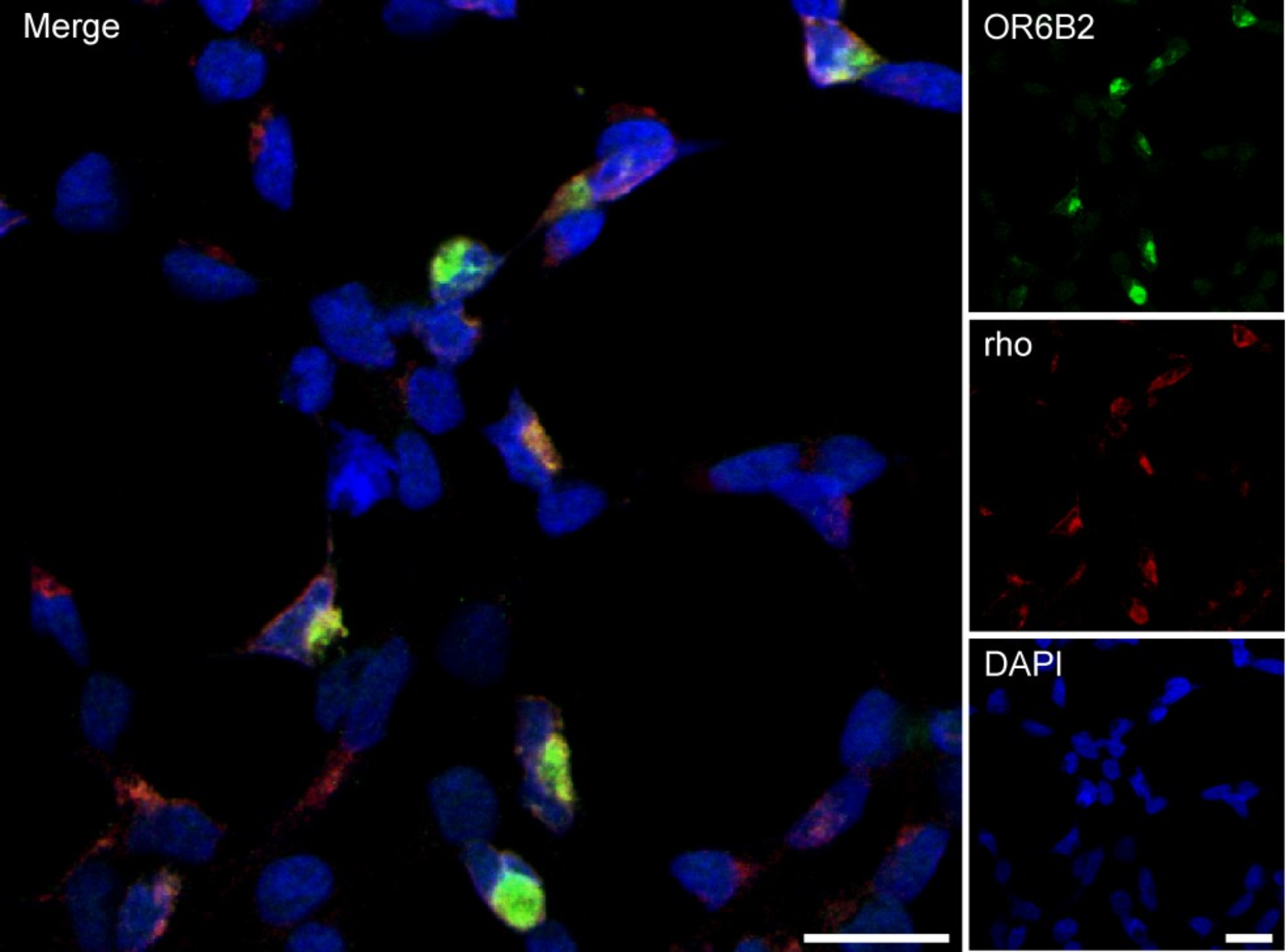

Supplement: S5 Fig — Immunostaining of Hana3A cells transiently transfected with OR6B2. The cells were stained with a specific OR6B2 antibody (OR6B2, green) and a rhodopsin-antibody (rho, red). DAPI staining (blue) was used to confirm the number and localization of cell nuclei. Scale bars: 20 μm. (TIF) [file pone.0128951.s005.tif]

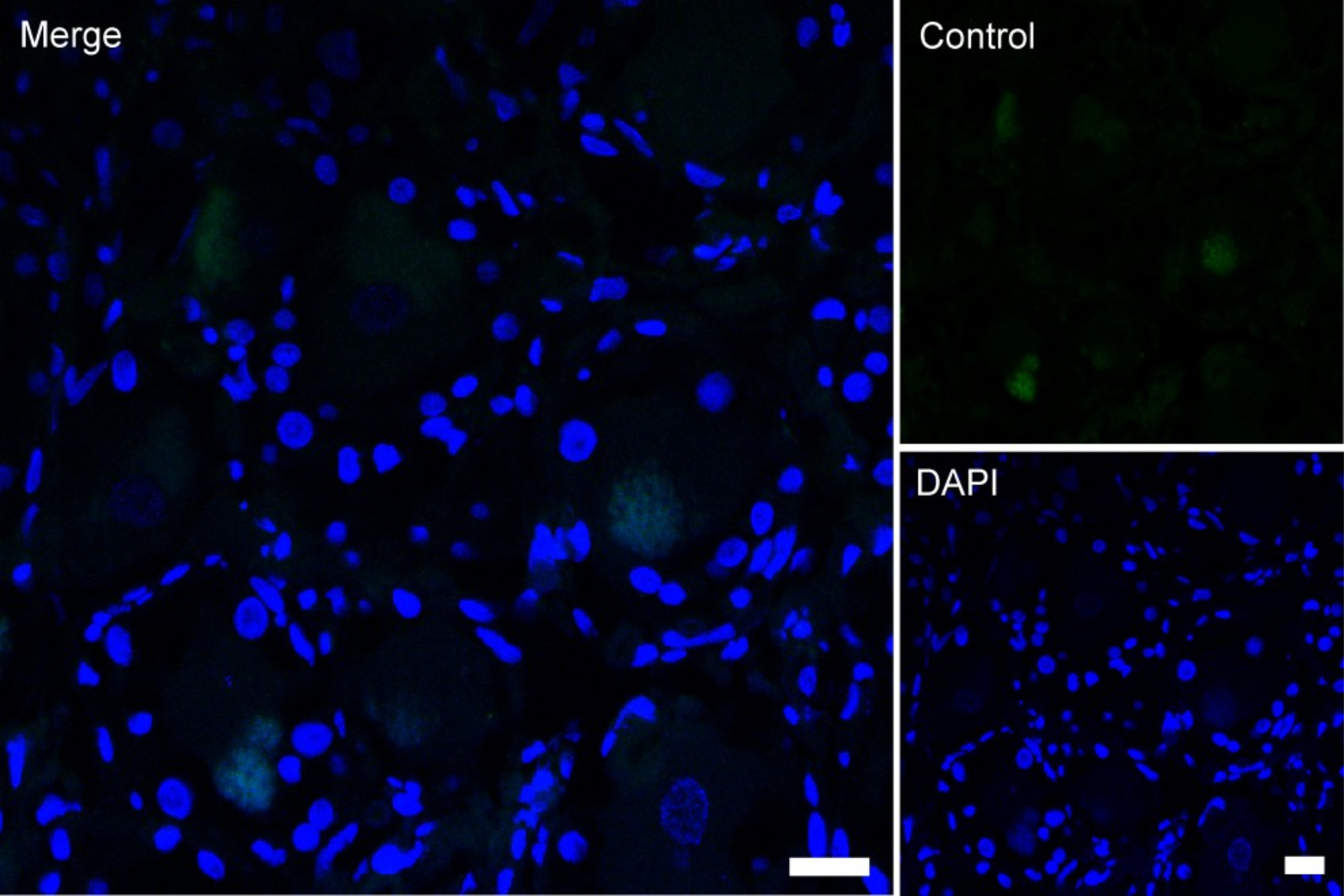

Supplement: S6 Fig — Control staining was performed without primary antibody, showing non specific staining by the secondary antibody (Alexa Fluor 488 Goat Anti-Rabbit, Control). Scale bar: 20 μm. (TIF) [file pone.0128951.s006.tif]

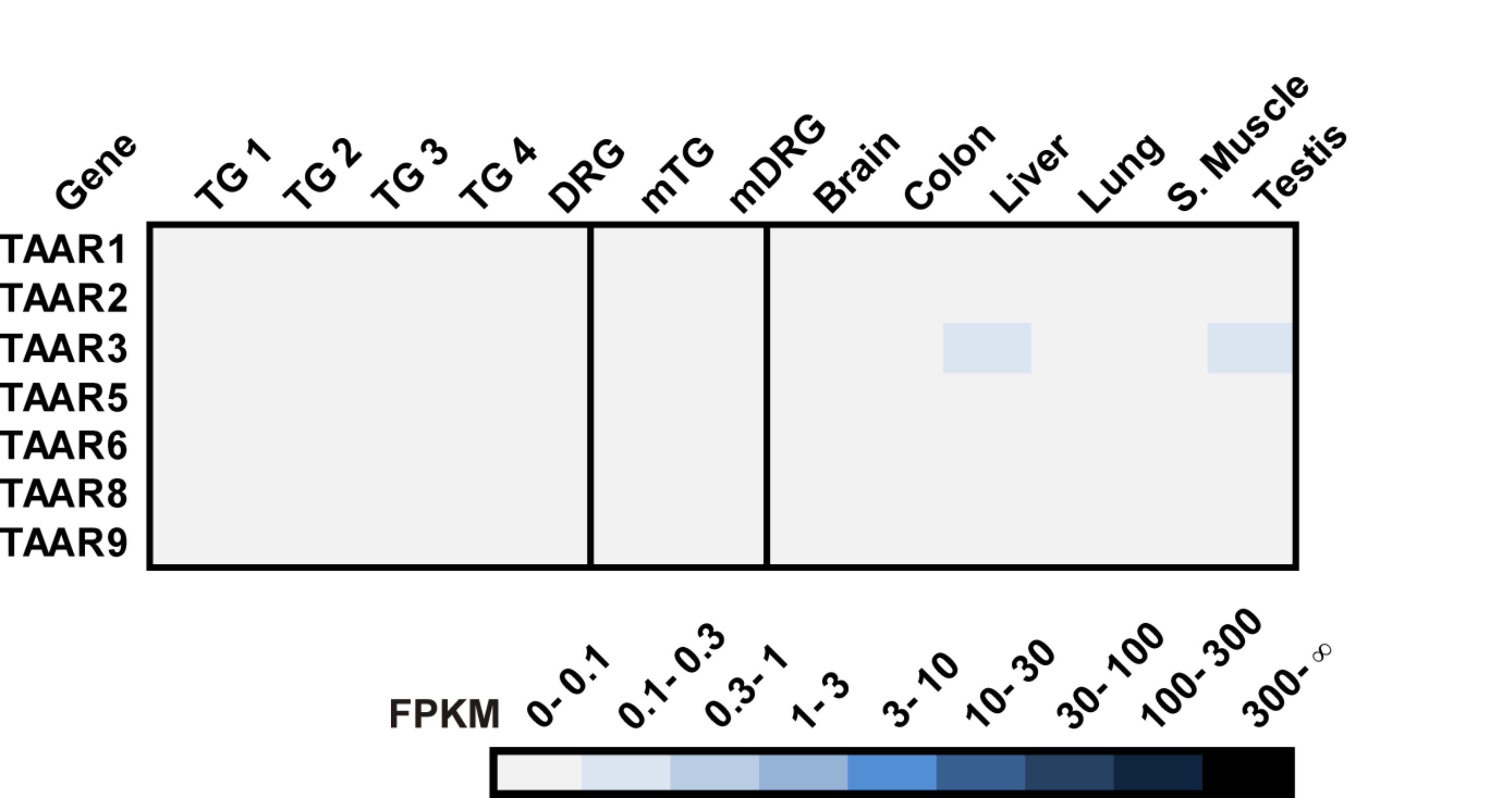

Supplement: S7 Fig — No TAAR transcripts could be detected in the sensory ganglia investigated. (TIF) [file pone.0128951.s007.tif]

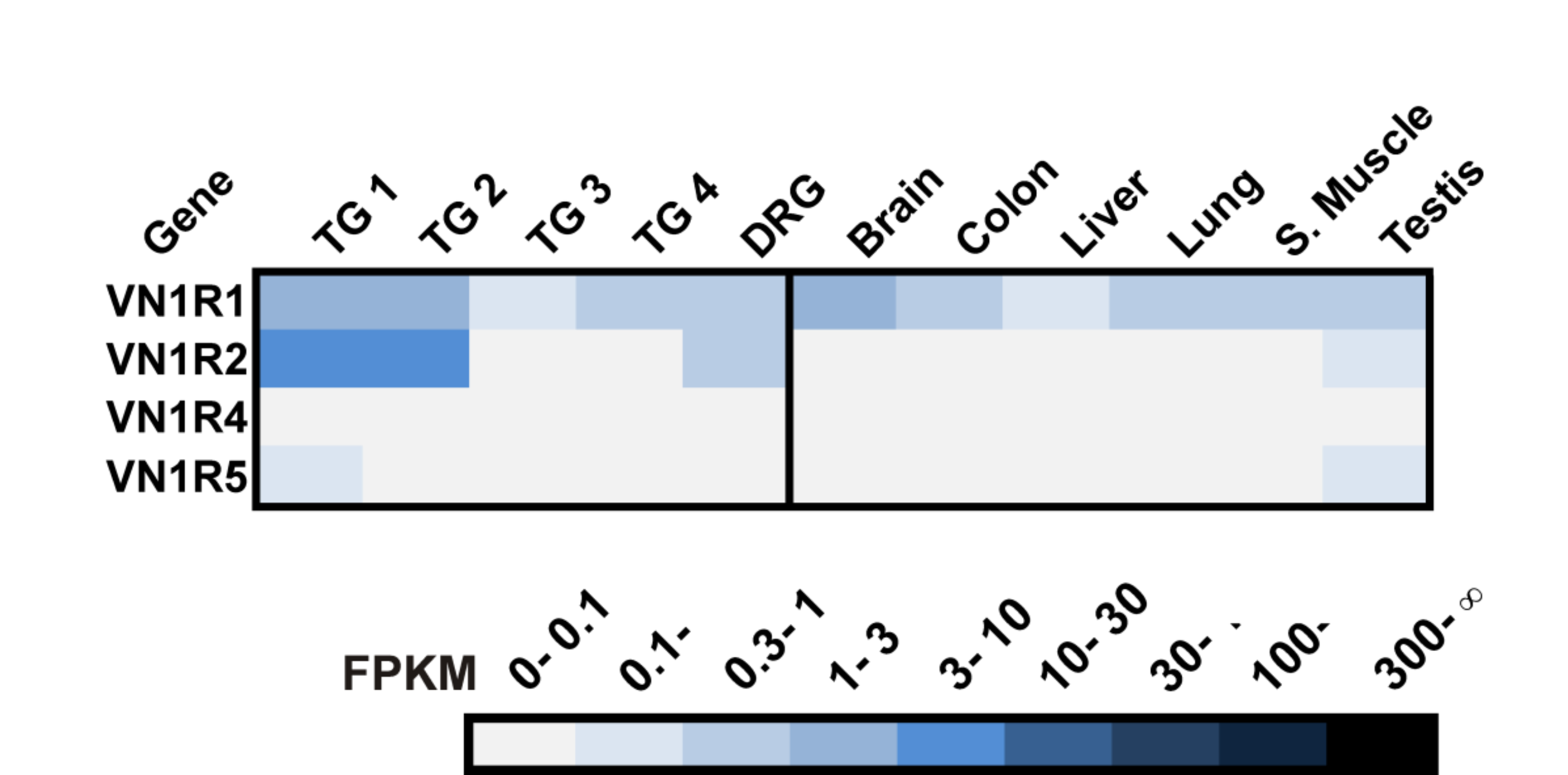

Supplement: S8 Fig — Shown are the FPKM values for VNR transcripts in sensory ganglia compared to different reference tissues (brain, colon, liver, lung, s. muscle, and testis). (TIF) [file pone.0128951.s008.tif]

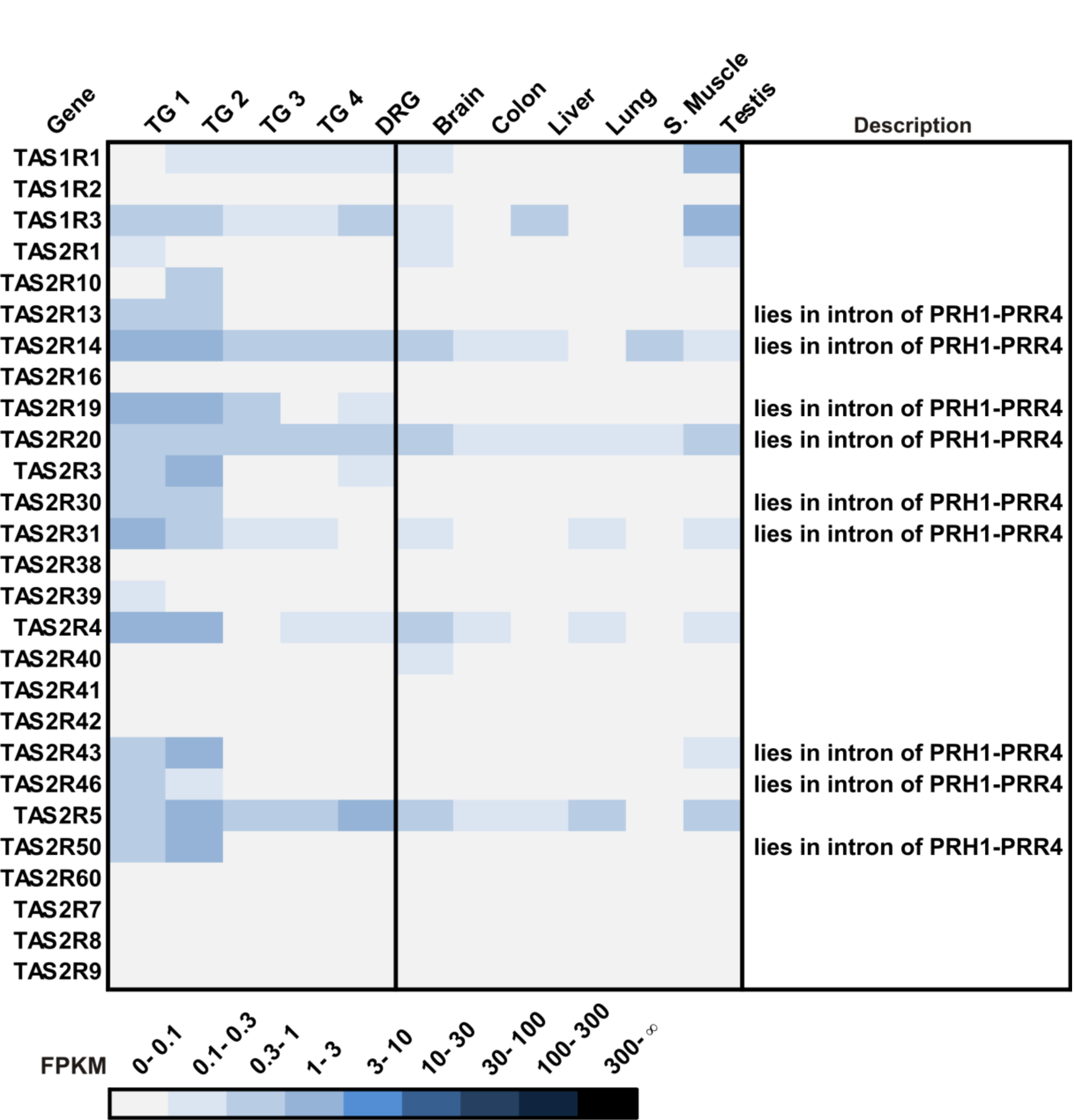

Supplement: S9 Fig — The FPKM values for TAS1Rs and TAS2Rs are shown. The sequencing read distribution was checked in the IGV, and in some cases, unclear expression in the human TG and DRG was revealed. Most of the detected TAS2R lay within introns in the moderately expressed PRH1-PRR4 gene. Intronic reads within the PRH1-PRR4 gene could stem from unprocessed transcripts of PRH1-PRR4 and not TAS2R transcripts as described in [155]. (TIF) [file pone.0128951.s009.tif]

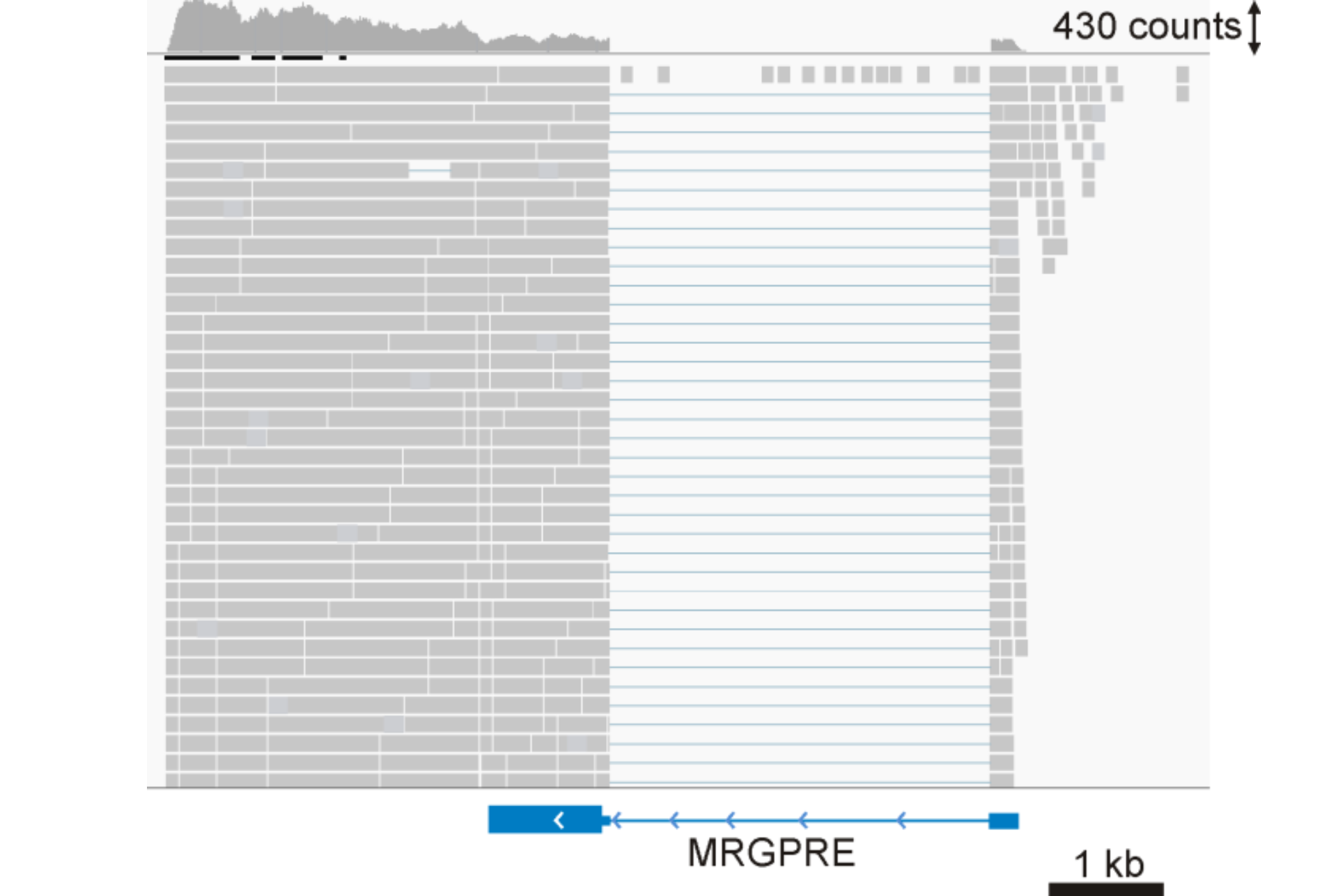

Supplement: S10 Fig — A large 3’UTR could be detected in transcripts from human MRGPRs (the example shown is MRGPRE), which is found in mice. (TIF) [file pone.0128951.s010.tif]

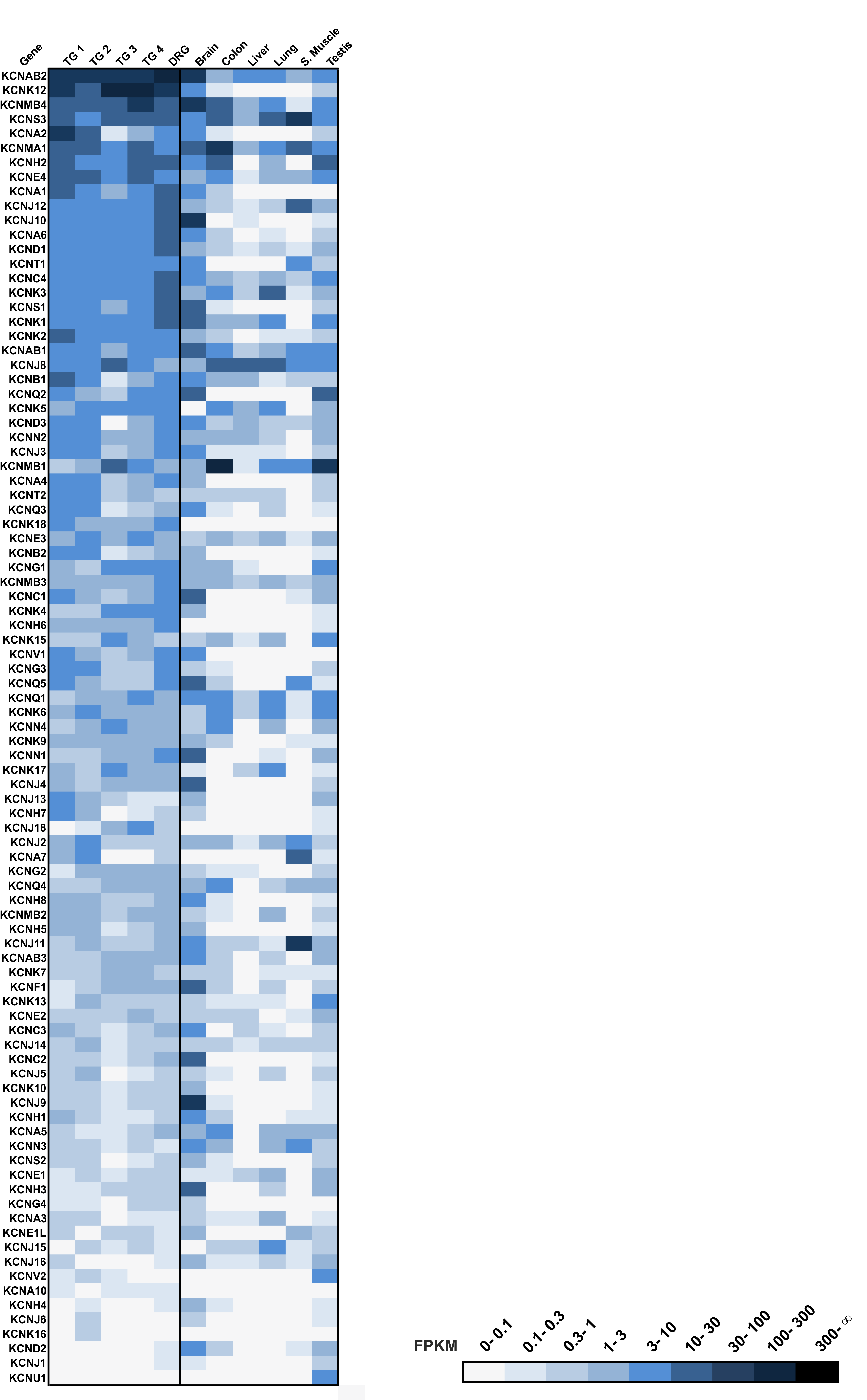

Supplement: S11 Fig — Genes are sorted by the mean of their expression values across all human sensory ganglia. (TIF) [file pone.0128951.s011.tif]

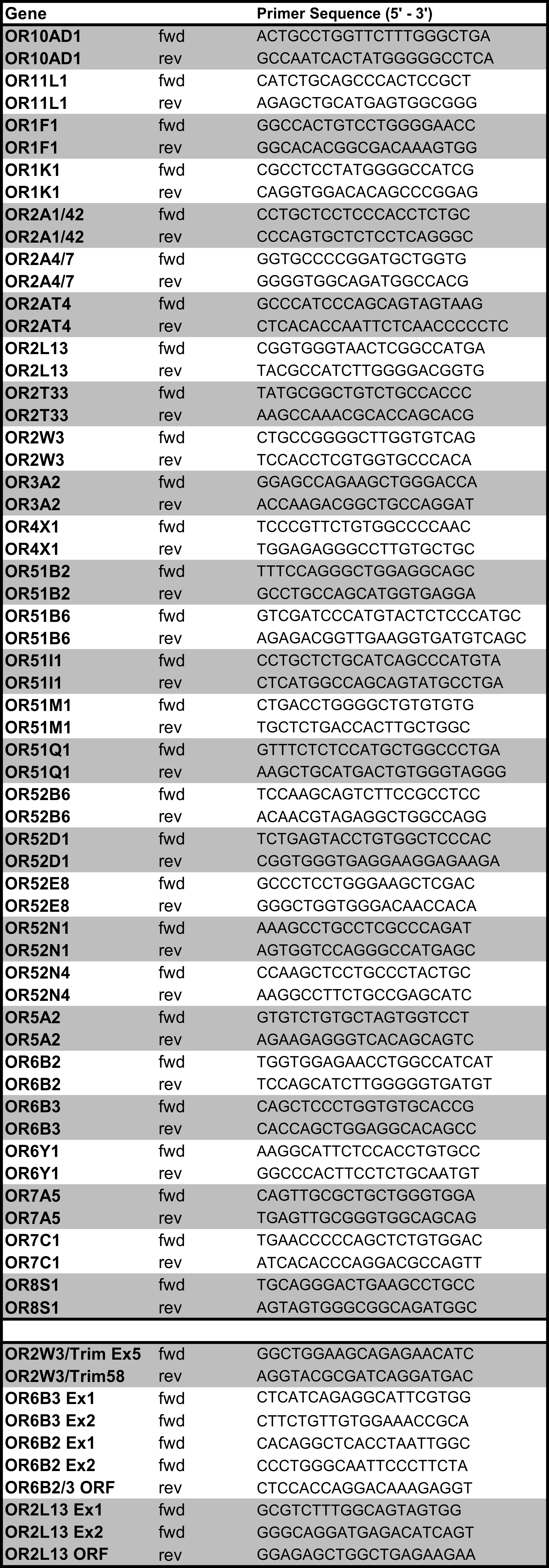

Supplement: S12 Fig — (TIF) [file pone.0128951.s012.tif]
